# Urinary antihypertensive drug metabolite screening using molecular networking coupled to high-resolution mass spectrometry fragmentation

**DOI:** 10.1007/s11306-016-1064-z

**Published:** 2016-07-05

**Authors:** Justin J. J. van der Hooft, Sandosh Padmanabhan, Karl E. V. Burgess, Michael P. Barrett

**Affiliations:** 1 Glasgow Polyomics, College of Medical, Veterinary and Life Sciences, University of Glasgow, Glasgow, UK; 2 Institute of Cardiovascular and Medical Sciences, College of Medical, Veterinary and Life Sciences, University of Glasgow, Glasgow, UK; 3 Wellcome Trust Centre for Molecular Parasitology, Institute of Infection, Immunity and Inflammation, College of Medical, Veterinary and Life Sciences, University of Glasgow, Glasgow, UK

**Keywords:** Antihypertensive drugs, Drug adherence, Drug metabolism, Fragmentation, High-resolution mass spectrometry, Metabolomics, Urine

## Abstract

**Introduction:**

Mass spectrometry is the current technique of choice in studying drug metabolism. High-resolution mass spectrometry in combination with MS/MS gas-phase experiments has the potential to contribute to rapid advances in this field. However, the data emerging from such fragmentation spectral files pose challenges to downstream analysis, given their complexity and size.

**Objectives:**

This study aims to detect and visualize antihypertensive drug metabolites in untargeted metabolomics experiments based on the spectral similarity of their fragmentation spectra. Furthermore, spectral clusters of endogenous metabolites were also examined.

**Methods:**

Here we apply a molecular networking approach to seek drugs and their metabolites, in fragmentation spectra from urine derived from a cohort of 26 patients on antihypertensive therapy. The mass spectrometry data was collected on a Thermo Q-Exactive coupled to pHILIC chromatography using data dependent analysis (DDA) MS/MS gas-phase experiments.

**Results:**

In total, 165 separate drug metabolites were found and structurally annotated (17 by spectral matching and 122 by classification based on a clustered fragmentation pattern). The clusters could be traced to 13 drugs including the known antihypertensives verapamil, losartan and amlodipine. The molecular networking approach also generated clusters of endogenous metabolites, including carnitine derivatives, and conjugates containing glutamine, glutamate and trigonelline.

**Conclusions:**

The approach offers unprecedented capability in the untargeted identification of drugs and their metabolites at the population level and has great potential to contribute to understanding stratified responses to drugs where differences in drug metabolism may determine treatment outcome.

**Electronic supplementary material:**

The online version of this article (doi:10.1007/s11306-016-1064-z) contains supplementary material, which is available to authorized users.

## Introduction

Mass spectrometry is pre-eminent in the analysis of drug metabolism. The enhanced sensitivity of new generation mass spectrometers including the high-resolution Orbitrap series of instruments (Zubarev and Makarov 2013) provides ever more capability to understand how drugs are metabolised by the human body. Mass spectrometry fragmentation (MS/MS or MS2) is widely used to find drug specific fragments (Levsen et al. 2005; Zhang et al. 2009; Gao et al. 2015) but the complexity of the data offers significant analytical challenges (Garg et al. 2015) and tools enabling advanced spectral analysis offer great utility in deriving new knowledge from this data (Hufsky et al. 2014; Ridder et al. 2014; Misra and van der Hooft 2016). Some of these tools will be discussed in the next paragraphs.

The mass defect filtering (MDF) approach is one example used to target drug derived metabolites within a complex extract (Zhu et al. 2006). The MDF approach uses drugs and core template filters and a set of commonly found transformations in drug metabolism such as hydroxylation, methylation, and decarboxylation and their calculated ‘mass defect shifts’, i.e., the fractional difference of the reactant and the product. With these filters and mass defects, potential drug metabolites can be found within a larger set of detected compounds in high-resolution mass spectrometry (HR-MS) data combined with data-dependent analysis (DDA) fragmenting the most abundant ions (i.e., the TopN ions) entering the mass spectrometer. More recently, the MS2Analyzer tool was developed to screen for specific product ions (mass fragments), neutral losses (difference between precursor ion and a product ion), and mass differences (difference between two product ions) in (HR-MS) fragmentation data (Ma et al. 2014). In principle, MS2Analyzer can be used to screen for spectra that contain pre-determined drug product ions, as well as commonly found losses caused by biotransformation of drugs, such as glucuronidation (i.e., 176.0321 Da) and sulfation (i.e., 79.9568 Da), which can be added into the search to aid in metabolite annotation.

Both of the above tools require specific user input to get meaningful results, including: (i) a list of expected drugs with their elemental formulas to determine their theoretical masses in the relevant ionization mode or (ii) mass spectral fragmentation data of drugs, whether experimental or in silico, to determine their specific product ions or neutral losses. This structural information is usually obtained from spectral libraries or previously characterised compounds. Currently, mass spectral libraries contain only a small fraction of the metabolites whose existence is known; for example, mzCloud (www.mzCloud.org) and MassBank (www.massbank.jp) contain fragmentation spectra of thousands of compounds, whereas PubChem (http://www.ncbi.nlm.nih.gov/pccompound) contains tens of millions of chemical structures (da Silva et al. 2015; Vinaixa et al. 2015) and many other compounds have yet to be catalogued in any database. Several computational tools that predict metabolite structures and fragmentation patterns in silico are in development (Hufsky et al. 2014; Ridder et al. 2014), but most are currently not capable of global analyses or comparison of large numbers of fragmentation spectra.

There is generally no a priori knowledge of all metabolite classes that will appear in untargeted metabolomics experiments, and in an era of precision and stratified medicine, comparing metabolic potential between individuals is of increasing importance. Therefore, tools that can compare and visualize large amounts of spectral data in an unbiased manner, i.e., without prior structural information, are needed. For example, molecular networking performs clustering of similar MS2 spectra from natural product extracts (Watrous et al. 2012; Yang et al. 2013). The tool compares MS2 fragmentation spectra in an unsupervised manner using cosine scoring on vectorised MS2 spectra. As a result, similar spectra are combined in a network node represented by a consensus spectrum. Nodes showing a degree of cross-node similarity are then connected by edges again based on cosine similarity scoring. If one or a few of the nodes in the network have a match to spectral databases present within the GNPS environment (http://gnps.ucsd.edu), such as MassBank (Horai et al. 2010), this can aid the structural annotation of closely associated nodes based on spectral similarity (Yang et al. 2013).

Few metabolomics studies have yet reported on the use of molecular networking combined with high-resolution metabolomics data to aid in spectral analysis of the large amount of spectral information resulting from data-dependent fragmentation. In this study, we used human urine extracts from a clinical cohort that encompassed patients who were receiving combinations of antihypertensive drugs comprised of different drug classes, including calcium channel blockers, ACE inhibitors, and beta blockers. The available meta-data was used to select urine extracts of 26 patients taking two or more different antihypertensive drug classes. The aim of this study was to examine if the combination of the molecular networking approach with (HR-MS) metabolomics fragmentation data would enable detection and visualization of clusters of antihypertensive and other drug related metabolites from human urine extracts.

The data shows that multiple drugs and a range of associated metabolites can be found in urine with no a priori knowledge of their presence. In addition, endogenous urine metabolites also form several clusters. The combined analysis offers new ways to assess the presence and metabolism of drugs in individual patients, and the influence of the patient’s metabolome on drug metabolism and the drug treatment outcome.

## Materials and methods

### Materials

#### Urine samples

Urine samples from anonymized human volunteers were used from a clinical sample set in the Glasgow Polyomics archive. These samples were obtained as part of a trial for which ethical approval was applied for through the Multi-centre Research and Ethics Committee (MREC), which was granted by the Scottish MREC and (with MREC N°06/MRE00/106). Informed consent was obtained from all individual study participants. Spot urine samples were obtained from the cohort of elderly hypertensive patients upon their first admission in the clinic. Urine extracts of 26 patients were selected as follows: diagnosed with hypertension, taking in a variety of different antihypertensive drugs (i.e., different drug classes), and availability of the sample extract in the Glasgow Polyomics archive. The resulting subject’s age range spanned from 42 to 87; 15 were male, 11 female; 4 were smokers; 5 were reported to have diabetes; and each reportedly took from 2 to 7 different classes of antihypertensive drugs. All recorded details of the patients can be found in the Supplementary Information (Supplementary Table S1).

#### Chemicals

HPLC-grade methanol, acetonitrile, isopropanol, and analytical reagent grade chloroform were acquired from Fisher Scientific, Loughborough, UK. HPLC grade H_2_O was purchased from VWR Chemicals, Fountenay-sous-Bois, France. Formic acid (for mass spectrometry) and ammonium carbonate were acquired from Fluka Analytical (Sigma Aldrich), Steinheim, Germany.

### Methods

#### Urine sample preparations

A general metabolome extraction procedure was performed (Creek et al. 2011): (i) 5 µL urine was extracted in 200 µL chloroform/methanol/water (1:3:1) at 4 °C; (ii) then vortexed for 5 min at 4 °C; (iii) then centrifuged for 3 min (13,000 g) at 4 °C. The resulting supernatant was stored at −80 °C until analysis. A pooled aliquot of the 26 selected urine samples was prepared prior to the LC–MS runs with DDA applying higher collision dissociation (HCD)

#### Analytical platform

A Thermo Scientific Ultimate 3000 RSLCnano liquid chromatography system (Thermo Scientific, CA, USA) was used. That system was coupled to a Thermo Scientific Q-Exactive Orbitrap mass spectrometer equipped with a HESI II interface (Thermo Scientific, Hemel Hempstead, UK). Thermo Xcalibur Tune software (version 2.5) was used for instrument control and data acquisition.

#### LC settings

The HILIC separation was performed with a SeQuant ZIC-pHILIC column (150 × 4.6 mm, 5 µm) equipped with the corresponding pre-column (Merck KGaA, Darmstadt, Germany). A linear biphasic LC gradient was conducted from 80 % B to 20 % B over 15 min, followed by a 2 min wash with 5 % B, and 7 min re-equilibration with 80 % B, where solvent B is acetonitrile and solvent A is 20 mM ammonium carbonate in water. The flow rate was 300 μL/min, column temperature was maintained at 25 °C, injection volume was 10 μL, and samples were maintained at 4 °C in the autosampler (Creek et al. 2011).

#### MS and MS/MS settings

*Positive negative ionization combined fragmentation mode* a duty cycle consisted of a full scan in positive ionization mode, followed by a TopN MS/MS (MS2) data dependent fragmentation event, taking the 10 most abundant ion species not on the dynamic exclusion list, followed by the same two scan events in negative ionization mode. Data acquisition was carried out in positive (+) and negative (−) switching ionization mode, using m/z 74.0964 (+) (ACN cluster), 88.07569 (+) (contaminant), and m/z 112.98563 (−) (Formic Acid cluster) as lock masses. The set up was calibrated [Thermo calmix (Pierce™ calibration solutions from Thermo Scientific), with additional masses at lower m/z; 74.0964 m/z (+) and 89.0244 (−)] in both ionization modes before analysis and a tune file targeted towards the lower m/z range was used.

Unless specified differently, full scan (MS1) data was acquired in both ionization modes in profile mode at 35,000 resolution (at m/z 200) using 1 microscan, an AGC target of 10^6^ cts, a maximum injection time of 120 ms, with spray voltages +3.8 and −3.0 kV, capillary temperature 320 °C, sheath gas flow rate 40, auxiliary gas flow rate 15 a.u., sweep gas flow rate 1 a.u, and a full scan mass window of 70–1050 m/z.

MS/MS (MS2) data was acquired in profile mode at 35,000 resolution using 1 microscan, an AGC target of 1 × 10^5^ cts, a maximum injection time of 120 ms, a loop count of 10, a MSX count of 1, a TopN of 10, an isolation window of 1.0 Da, an isolation offset of 0.0 Da, a stepped normalized collision energy (NCE) (HCD) mode combining 25.2, 60.0, and 94.8 NCEs into one fragmentation scan, an underfill ratio of 20 %, an intensity threshold of 1.7 × 10^5^ cts, and the dynamic exclusion was set to 15 s. Further settings were: no apex trigger, no charge exclusion, peptide match was off, exclude isotopes was on, and if idle, the setting ‘the machine does not pick up other ions’ was chosen.

*Positive or negative ionization separate fragmentation modes* as for the combined experiments, with the following modifications: full scan (MS1) resolution (at m/z 200) was set to 70,000, MS/MS (MS2) resolution (at m/z 200) was set to 17,500, MS/MS maximum injection time was set to 80 ms and the underfill ratio set to 10 %, with a resulting intensity threshold of 1.3 × 10^5^ cts. The duty cycle consisted of one full scan (MS1) event and one Top10 MS/MS (MS2) fragmentation event.

*Positive negative ionization combined full scan mode* as for the combined experiments, with the following modifications: full scan (MS1) resolution (at m/z 200) was set to 70,000. The duty cycle consisted of two full scan (MS1) events.

### Data acquisition and processing

#### Data acquisition

Blank runs, quality control samples (beer and serum extracts in accordance with standard procedures at Glasgow Polyomics) to assess the performance of the mass spectrometer in terms of chromatography and mass intensities, and three standard mixes containing 150 reference compounds were run to assess the quality of the mass spectrometer and to aid in metabolite annotation and identification (Creek et al. 2011). The pooled sample was run prior to and across the batch every 6th sample to monitor the stability and quality of the LC–MS run, whereas the samples were run in a randomized order.

Immediately after acquisition, all raw files were converted into mzXML format, thereby centroiding the mass spectra and separating positive and negative ionization mode spectra into two different mzXML files using the command line version of MSconvert (ProteoWizard). Accurate masses of standards were obtained well within 3 ppm accuracy and intensities of the quality control samples (a beer extract and a serum extract) were within specifications.

All 26 urine extracts were run in the combined fragmentation mode, a subset of 12 urine extracts underwent the separate fragmentation modes collecting fragmentation data for both modes in two separate files, and a subset of 6 urine extracts were run in combined full scan mode using three replicate injections. A number of separate fragmentation mode files were run as part of another batch; however, only small retention time drifts were observed (within 0.15 min) and the comparison does not use retention time information, as it is based on MS2 spectral similarity. See Supplementary Table 1 for detailed information where it is specified for each urine extract which modes were recorded.

#### Data processing

**Fig. 1:**
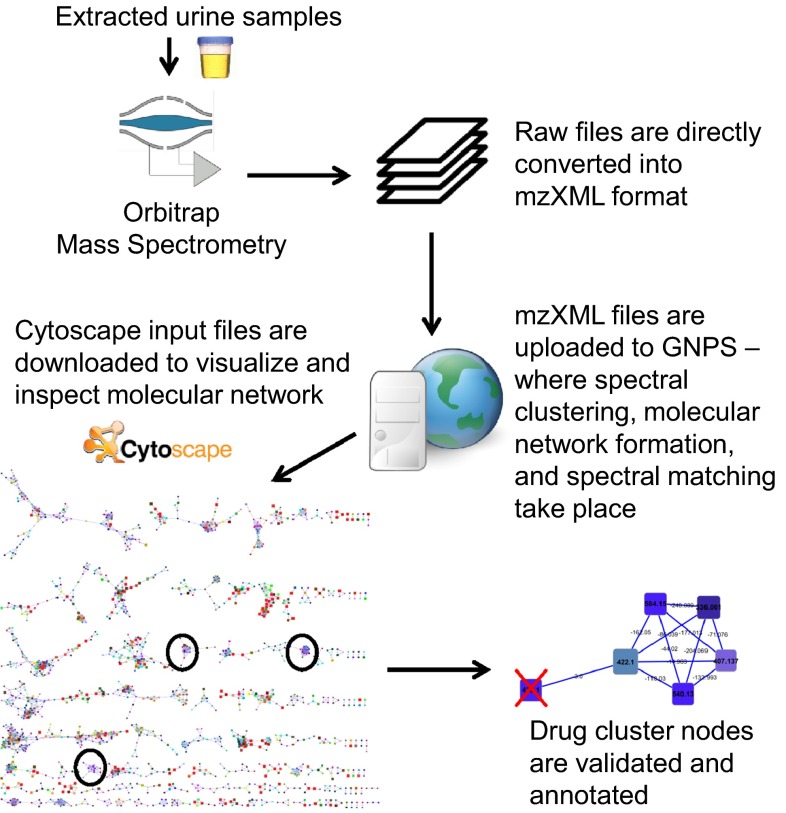
Flowchart explaining the different steps in data generation, processing, and analysis to enable the annotation of drug clusters and drug metabolites from a molecular network obtained from urine extracts

The data processing and data analysis steps are summarized in a flowchart (see Fig. 1).The mzXML files were uploaded into the Global Natural Products Social Molecular Networking (GNPS) environment (http://gnps.ucsd.edu—a free account is needed to log in) using an FTP server (FileZilla, version 3.10.1.1). Parameter optimization for molecular network generation for the (HR-MS) data sets resulted in the following settings. All MS2 spectra present in the data were clustered with MS-Cluster with a so-called ‘parent mass tolerance’ of 0.25 Da and a MS/MS fragment ion tolerance of 0.005 Da to create consensus spectra. Then, consensus spectra that contained less than 2 spectra were discarded. A network was created where edges were filtered to have a cosine score above 0.55 and 2 or more matched peaks. Further edges between two nodes were kept in the network only if each of the nodes appeared in each others respective top 10 most similar nodes. The spectra in the network were then searched against the GNPS spectral libraries. The library spectra were filtered in the same manner as the input data. All matches kept between network spectra and library spectra were required to have a cosine score above 0.6 and at least 4 matched peaks. Analog search was enabled against the library with a maximum mass shift of 100.0 Da. All parameters and their values used can be found in Supplementary Table S2. Running times were under 15 min for both combined and single mode fragmentation files. Cytoscape, network visualization software (http://www.cytoscape.org/), was then used to further process and visualize the downloaded molecular network data. The recommended graphical layout style is FM3 which is available for Cytoscape versions 2.8.1 and below. Thus, the molecular network was uploaded into Cytoscape (version 2.8.1) following the documentation available on the GNPS website. After applying the FM3 layout plugin, the molecular network was saved in.cys format (Cytoscape Session File) and reopened in Cytoscape version 3.2.0, where labelling and colouring of nodes and edges was conducted. Most importantly, the nodes were labelled with precursor masses, and coloured such that two nodes have the same colour when they are present in the same set of files (using the rainbow pallet), and accordingly, two nodes having similar colours means that they are present in a similar set of files, often differing in one or two files). Finally, the size of the nodes was made proportional to the number of unique files from where the node spectra originated, i.e., the larger the node, the more unique files its spectra originated from. The edges were labelled with the mass differences between the two nodes they connect. The resulting molecular networks for the combined and separate fragmentation modes were then inspected in the Cytoscape environment.

### Data analysis

#### Molecular network inspection

The first step of the molecular network analysis was to identify drug related clusters, which we define as subsets of connected nodes in the molecular network that all relate to one parent drug (or endogenous compound class in case of non-drug compounds). The resulting molecular network was checked by initial library matches to the spectral reference data present in the databases within the GNPS environment. Clusters containing drug annotations were then selected and the individual nodes and edges were inspected, as well as the cluster topology.

#### Cluster annotation

These drug related clusters were annotated using the library matching of GNPS to MassBank and the FDA_library as a starting point, resulting in so-called ‘seed nodes’ (Yang et al. 2013). If a drug related metabolite was matched from any of the libraries present in the GNPS environment, the cluster was further inspected. Clusters with a topology similar to the seven ‘GNPS-annotated drug clusters’ were annotated using spectral search in MzCloud of the most abundant MS2 spectrum belonging to the node represented in the highest number of different urine extracts. In addition, MAGMa was used to generate potential candidates if no successful spectral match was found. Endogenous urinary metabolite networks were annotated based on ‘seed node annotations’ or spectral matching of node spectra to MzCloud. Since the primary aim of this manuscript was to examine drug metabolites in the urine extracts, no further individual annotations on endogenous clusters were conducted.

#### Validation of molecular nodes

A node in the molecular network is a set of MS2 spectra each with a cosine similarity score of 0.95 or higher to each other (Yang et al. 2013) hence, this means that spectra of several isomers or very related compounds could be combined in one node. If such a situation occurs, the retention time associated with the node will become the average RT of the different MS2 spectra associated and as a result will have a relatively large retention time deviation recorded for it. In addition, nodes can represent isotopes, in-source fragments, or adducts of ‘real metabolites’, as no feature grouping is currently performed during the molecular networking. Thus, the nodes in the cluster need to be validated in the raw data by checking the number of metabolites represented by the node. Additionally, the most likely elemental formula was assigned and a theoretical mass was determined. Then, based on the validated metabolite spectra, recurring product ions were noted (see Supplementary Table 4) and used to mine the urine extracts by extracted ion chromatograms of those fragment masses. If they co-occurred in the spectrum, the precursor ion mass was checked against the list of already annotated metabolites–and added if it was absent. All drug related clusters prior to validation are presented in the Supplementary Information (see Sect. 2 in Supplementary Materials, Supplementary Figs. S2, S3, S4, S5, S6, S7, S8, S9, S10, S11, S12, S13, S14, and S15); in the other figures the nodes representing non-drug metabolites (e.g. isotopes or fragments of other nodes) were omitted from the cluster and colours and labelling were adapted to improve readability.

#### Metabolite annotation

The drug metabolites were annotated using the library matching, MzCloud database, and by searching for existing drug spectra in Massbank of North America (MoNa, http://mona.fiehnlab.ucdavis.edu/#/). Metabolite annotations are reported according to the Metabolomics Standards Initiative (MSI) metabolite identification (MI) levels: (1) for unambiguously identified, (2) for a spectral or literature match, (3) for a metabolite classification, (4) for metabolites that can be characterized by a retention time, mass, and fragmentation spectra if available (Sumner et al. 2007). Thus, if a reliable spectral match was found, the identification level was recorded as 2, if no match was found but a plausible elemental formula, drug specific product ions, and a likely annotation could be assigned, e.g., hydroxylated form of parent drug, the identification level was put to 3. MSI MI level 4 was given to those metabolites where the drug specific product ions were present, but no likely elemental formula or annotation could be assigned, or where the recurring product ions could not be assigned to a unique drug-related core structure metabolized by the human body. All structural information obtained was recorded in Supplementary Table S3.

## Results

### Molecular networking to discover drug related metabolite clusters

Unsupervised large-scale comparison of (HR-MS) fragmentation spectra was conducted on data files from urine extracts suspected to contain antihypertensive drug metabolites. The resulting networks were analysed to find potential drug metabolite clusters using the annotation based on library matching within the GNPS environment. Seven clusters contained one or more annotated nodes with drug related compounds (see Table 1), with some clusters containing several annotated nodes (indicated in Table 1 with ‘Total annotated nodes’) accumulating to a total of 16 drug related nodes annotated by spectral matching. Six of those drugs are known antihypertensives. Further inspection of the annotated nodes revealed that four of them were correctly matched to a metabolite with the correct mass and fragmentation spectrum (indicated in Table 1 as ‘correctly annotated’). The other twelve annotated nodes were partial matches where the mass of the matched metabolite did not correspond to the mass of the fragmented metabolite, while parts of their fragmentation spectra did show high similarity (indicated in Table 1 as ‘related compound’).

**Table 1:** Number of GNPS annotated nodes in each drug related cluster, with ‘Parent drug’ representing the annotated parent drug, ‘total annotated nodes’ showing how many nodes had a database hit, ‘correctly annotated’ indicating how many nodes were indeed matched to the correct drug metabolite (i.e., mass and fragmentation pattern fitted), and ‘related compound’ means a structurally related drug metabolite was matched from the in GNPS present fragmentation libraries such as Massbank

Parent drug	Total annotated nodes	Correctly annotated	Related compound
Clodipogrel	1	1	0
Irbesartan/losartan	3	1	2
Verapamil	3	1	2
Atenolol/bisoprolol	2	1	1
Ranitidine	3	0	3
Metformin	3	0	3
Paracetamol	1	0	1
Total	16	4	12

**Fig. 2:**
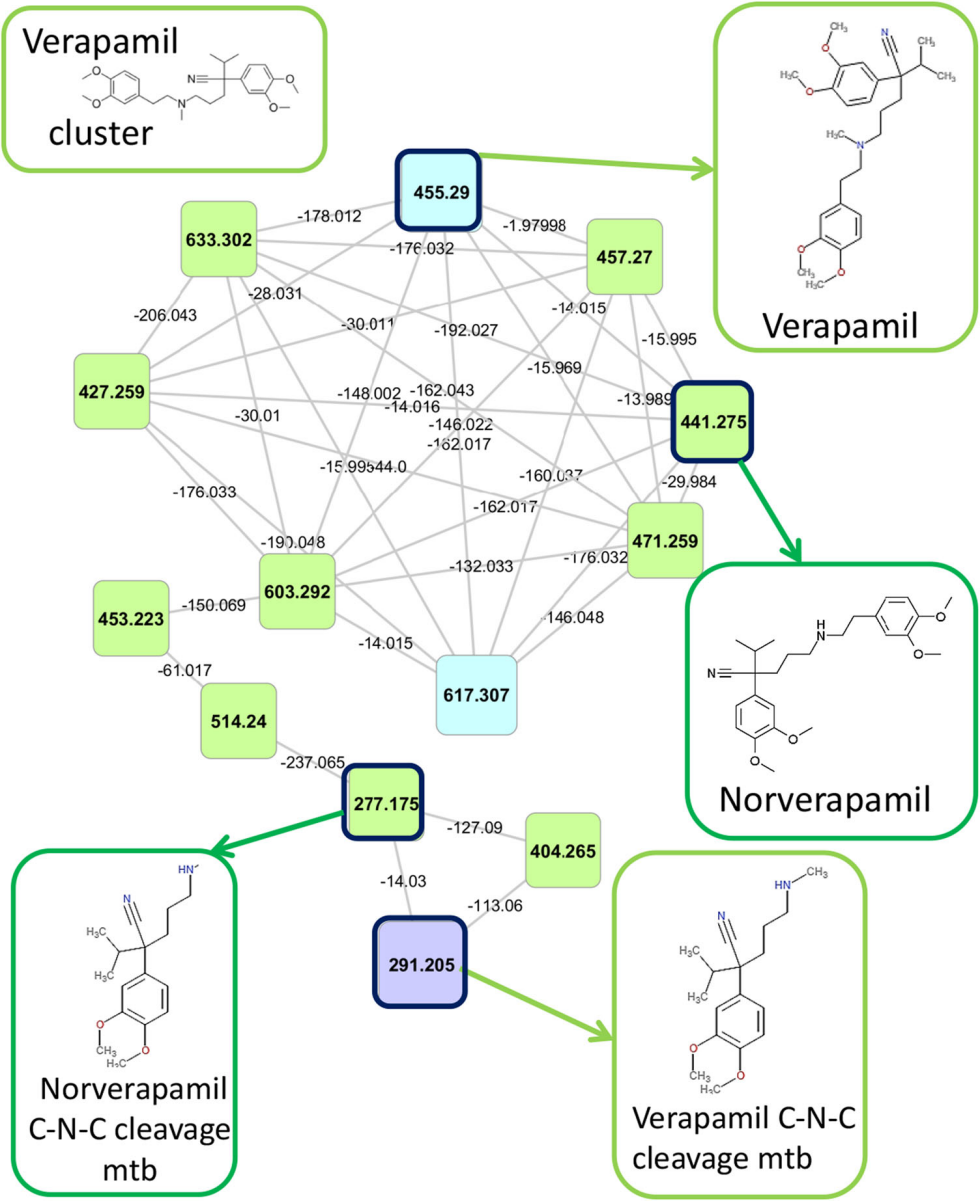
Verapamil related cluster: the cluster in the molecular network that contains verapamil metabolites. In total, 2 urine extracts contain one or more verapamil related metabolites. The four metabolites that could be annotated based on spectral matching, including verapamil itself, are highlighted in the cluster and structures are presented for each of them

Figure 2 shows the example of the ‘verapamil cluster’ that was identified since the GNPS annotation of three of its nodes included verapamil itself. The fact that this cluster has fourteen nodes indicates extensive biotransformation in man. Two previously described C–N–C cleavage metabolites of verapamil (Eichelbaum et al. 1979) could be matched to spectral data available in MassBank, hence the four highlighted nodes in Fig. 2 are MSI MI confidence level 2 (see Sect. 2.4.4). The other nodes were annotated using mass differences to the seed nodes (i.e., the annotated nodes), which included 176.032 (typical for glucuronidation), 14.015 (typical for methylation), and 16.000 (typical for hydroxylation), as well as manual inspection of their fragmentation spectra. Subsequently, key drug-related fragments were determined based on spectral comparisons (see Supplementary Table S4). The key fragments were then used to mine the fragmentation files for more related drug metabolites omitted by the clustering approach; in some cases resulting in the additional annotation of drug metabolites (see Supplementary Table S5).

The verapamil metabolites found by the molecular networking show a variety of structures ranging from glucuronides, typically larger-sized metabolites, to cleavage products, typically smaller-sized metabolites, also eluting at different retention times. Furthermore, it is interesting to note that seven nodes show high interlinkage in the verapamil cluster, indicating that they share a common substructure causing many edges to interlink the nodes. This was also observed for the other drug related clusters in Table 1 (see Sect. 2 in Supplementary Materials, Supplementary Figs. S2–S15 or the Cytoscape session file, MolecularNetworks_DrugClusters.cys, in the Supplementary Materials). Furthermore, several different drugs also cluster together, for example losartan and irbesartan which relates to their sharing of the sartan substructure that produces overlapping fragmentation spectra (Lo et al. 1995; Chando et al. 1998).

**Fig. 3:**
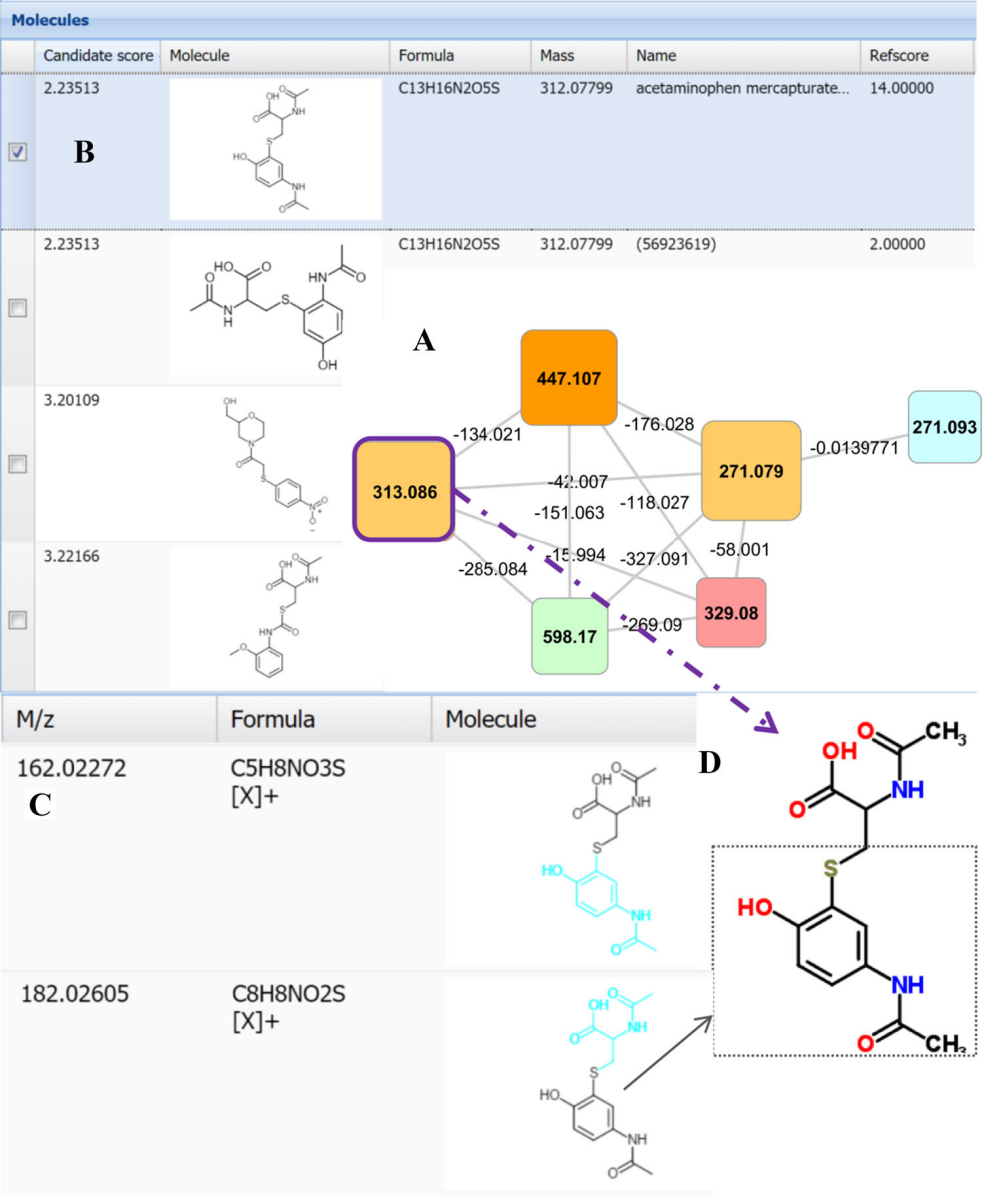
MagMa screenshot displaying the annotation of node 313.066 in highly interlinked cluster (**a**); **b** the fragmentation pattern matched paracetamol mercapturate (conjugate of paracetamol and *N*-acetylcysteine), which is the top ranked hit in the results. **c** Fragment annotation by MagMa, the fragment C_8_H_8_NO_2_S is also highlighted in the structure of paracetamol mercapturate (**d**), encompassing the paracetamol part of the conjugate including the sulphur atom from *N*-acetylcysteine

Suspected additional drug related clusters were recognized by their highly interlinked topology, and subsequently annotated by spectral matching (i.e., to MzCloud, www.mzcloud.org; or literature reference tables) or by using the fragmentation annotation software MAGMa (Ridder et al. 2013) (see the cluster shown in Fig. 3). Analysis of the metabolites present in all 11 urine extracts containing spectra of this cluster (the nodes with m/z 313.086 and 271.079 ([M+H]^+^)) resulted in the top-ranked candidate structure of paracetamol-mercapturate (conjugate of paracetamol and *N*-acetylcysteine) for m/z 313.086—a metabolite of a non-antihypertensive drug. Further inspection of the fragment annotation revealed key fragments of paracetamol-mercapturate-like metabolites as shown for the C_8_H_8_NO_2_S fragment in Fig. 3c, d where a diverse set of sulphur containing paracetamol conjugates were annotated. The urines that contain these metabolites also contained paracetamol-*O*-sulphate and paracetamol-*O*-glucuronide confirming paracetamol use by these patients.

The molecular networking approach thus allows annotation of numerous antihypertensive drugs (and antidiabetic and histamine antagonists, see Sect. 3.3) in the urine extracts. Moreover, we observed a high interlinkage topology for these drug related clusters.

### Molecular networking to analyse drug metabolite occurrences

**Fig. 4:**
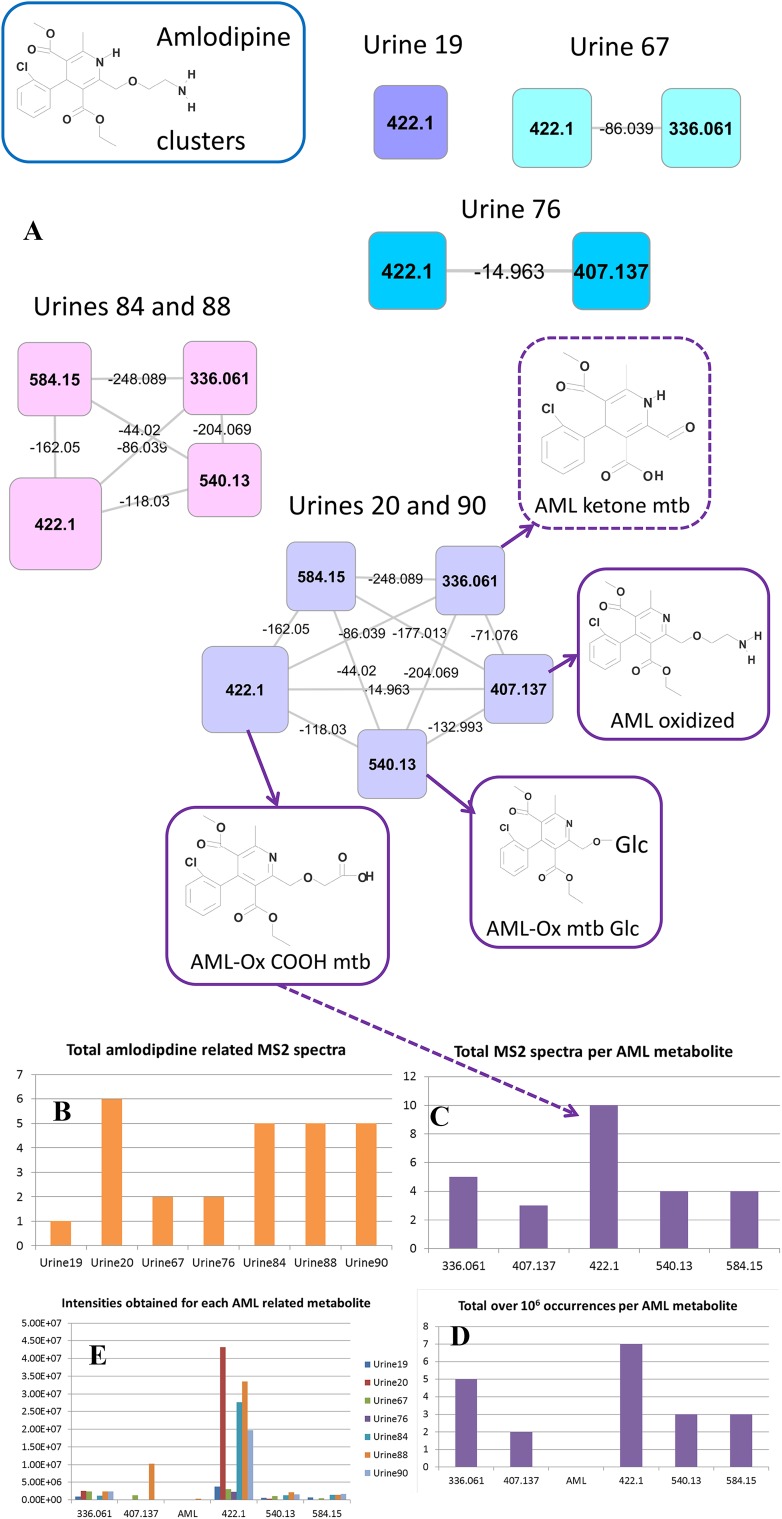
Amlodipine (AML) related clusters. The molecular networking and subsequent node validation process resulted in the AML-related clusters in (**a**). The structures for those metabolites that could be annotated by matching product ions to literature data are shown (*solid boxes*) and the annotation of a AML metabolite based on spectral homology and mass differences is also presented (*dashed box*), where mtb is short for metabolite, Glc for glucuronide, and Ox for oxidized. In total, 7 urine extracts contained one or more AML metabolites, here represented in five clusters based on the presence/absence of AML metabolites. Spectral occurrences were obtained from the molecular network, **b** presents the total number of AML-related spectra in each urine extract that was included in the cluster, **c** presents the total number of acquired MS2 spectra for each AML related metabolite, **d** total over 1 × 10^6^ occurrences for each AML metabolite, and **e** shows the intensities (cts) for each AML related metabolite extracted from the LC–MS full scans using accurate mass and retention time information obtained during the node validation process, including the accurate mass for the parent drug amlodipine. An *arrow* is drawn to connect the annotated oxidized amlodipine (AML-OX) COOH metabolite with the corresponding peak in plot C

The molecular networking approach also allows for quick comparison of the relative abundance of drug metabolites. Spectral occurrences in the amlodipine (AML) cluster (Fig. 4a) were extracted for detailed analysis. Three annotations in the cluster could be matched to data from the literature where standards for those metabolites had been synthesized (Suchanova et al. 2008). This spectral occurrence approach offers rapid interrogation of the data in a manner analogous to spectral counting applied in quantitative proteomics (Bantscheff et al. 2007). In Fig. 4b, we present the total number of AML-related MS2 spectra found in the seven urines in which any AML-related metabolites were found, and in Fig. 4c, the total MS2 spectra found for each AML-related metabolite are shown. Differences in abundance of the AML-metabolites were evident in different patients (Fig. 4c), and particular drug metabolites appear to be well suited for studies based on spot urine samples to ascertain patient compliance for taking the prescription drug given their universal appearance in patients while parent compound is not measured (AML itself was found in just one patient, and then in trace amounts) (Fig. 4d). Based on the AML-related MS2 spectra, the 422.1 node, containing the oxidized amlodipine carboxylic acid metabolite (see Fig. 4a), is the most readily detected metabolite. In order to check how well this MS2 spectral based analysis represents the actual LC–MS peak abundances, the accurate masses of the node metabolites were used to determine LC–MS peak intensities in the seven urine extracts. Figure 4d, e shows how the MS2 spectral counting does represent the information obtained from the LC–MS peaks and thus allows for quick interrogation of inter-sample differences.

Irbesartan and losartan are both angiotensin II receptor antagonists. Both of these parent drugs, and their metabolites, were clustered based on their common sartan [(1*H*-tetrazol-5-yl)biphenyl-4-yl] backbone (Fig. 4). The metabolism of irbesartan has been extensively studied using 14C-labelled parent drug to trace its metabolites in urine (Chando et al. 1998) and eight labelled irbesartan metabolites in addition to the parent drug itself were found. The authors used fragmentation data as well as 1D and 2D-NMR spectra to identify structurally those metabolites as completely as possible. Comparing their reported elemental formulas and nominal product ions, we could match two irbesartan metabolites with confidence (MSI MI level 2) based on the unique product ions for M1 (C_11_H_19_N_2_O_3_, nominal mass 227) and M6 (C_11_H_17_N_2_O_2_, nominal mass 209). The spectral match for three other metabolites was ambiguous (MSI MI level 3) and complementary structural information (such as from NMR) or availability of reference compounds would be necessary to fully identify those metabolites. Overall, 4 different masses matched between our analysis and that of Chando et al., where the co-eluting hydroxylated losartan metabolites underwent extensive preparative HPLC to separate them.

**Fig. 5:**
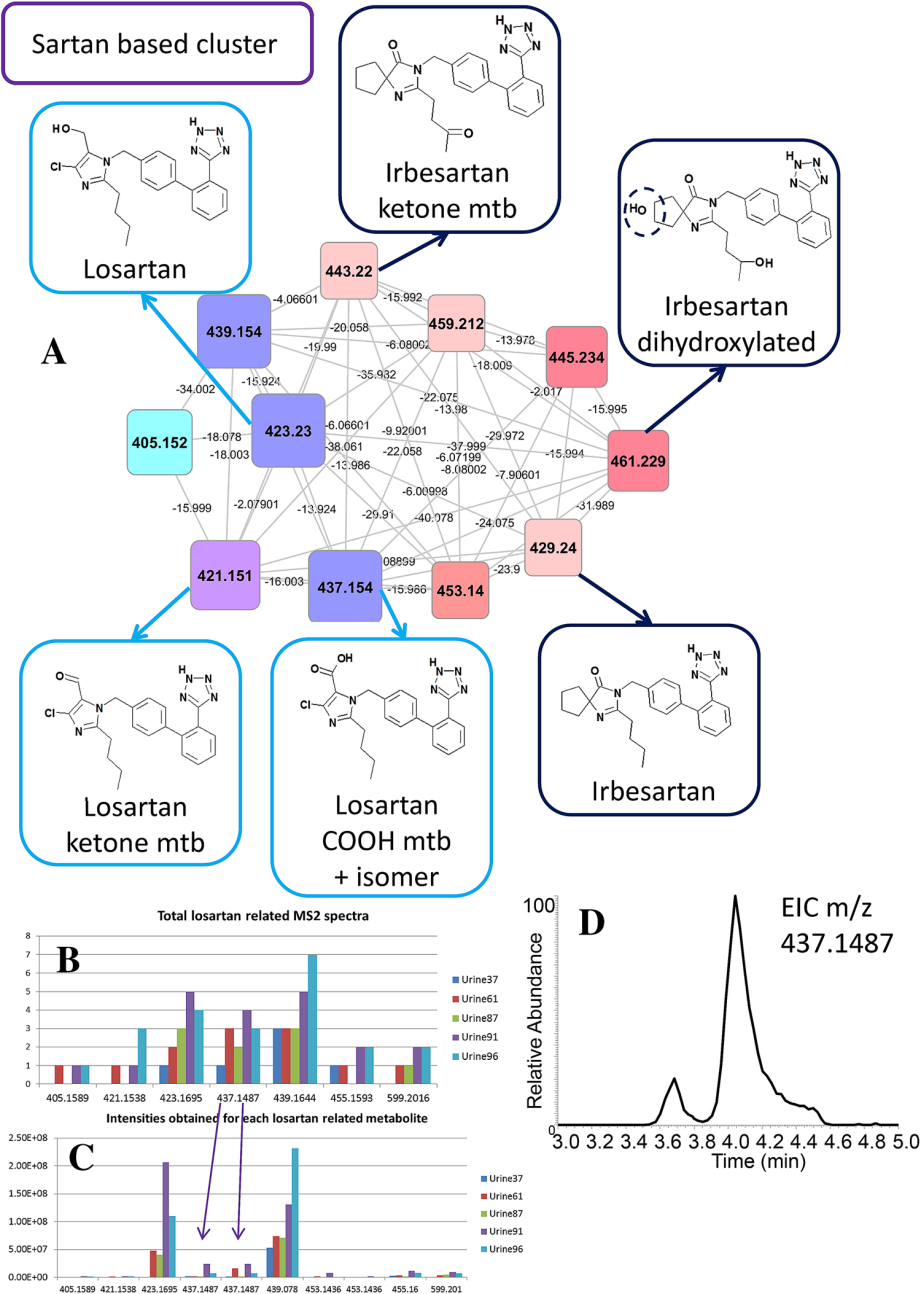
Sartan (irbesartan and losartan) related cluster. In total, 5 urine extracts contain 1 or more losartan metabolites and 1 unique urine extract contains irbesartan related metabolites. *Nodes* representing chlorine isotopes of losartan metabolites were removed from the cluster. **a** Three losartan and three irbesartan related metabolites are highlighted, annotated based on spectral matching or spectral homology and mass differences, where mtb is short for metabolite. b The total number of acquired MS2 spectra for each metabolite or metabolite group is shown. **c** The abundance (cts/s) of losartan metabolites across the different urine extracts. Note that the two 437.1487 isomers cause a relative high spectral counting (total number of MS2 spectra) for this metabolite group as a result of being combined in one node. **d** Extracted ion chromatogram for the m/z 437.1487, showing the two closely eluting isomers. In Supplementary Fig. 1, their two MS2 spectra are displayed

Losartan was found in five urine extracts. Its metabolites were easily discriminated from the irbesartan metabolites since the chlorine atom in their structure generates a characteristic isotope pattern in full scan mode. A report on losartan metabolism (Schmidt and Schieffer 2003) did not report on mass spectra, but we used the assigned elemental formulas typically containing six nitrogen atoms and one chloride atom, to allow matching to an active losartan metabolite EXP3174, and a ketone derivative of losartan (Fig. 5). Figure5b, c show the MS2 spectral occurrences and the abundances obtained from the LC–MS peaks. The parent drug and a hydroxylated derivative are abundant in all five urine extracts. Manual inspection of the losartan nodes further revealed two isomers of EXP3174 (a COOH metabolite, m/z 437.1487, [M+H]^+^), (Fig. 5d). The MS2 spectra of the two peaks showed differences in abundances for drug related product ions (see Supplementary Fig. S1).

Thus, manual inspection of the clusters and nodes remains important to ensure the presence of one or several metabolites in one node. The drug metabolism information obtained through metabolite networking also warrants further investigation on the metabolism of drugs like losartan to establish the potential role of the structurally differential isomers. From Fig. 5, it can be observed that both the parent drug and a hydroxylated form of losartan can be used in drug adherence studies, as these are the most abundant and widely present losartan metabolites detected in this set of patients.

### Unsupervised clustering also reveals other drug types and endogenous metabolites in urine

The unsupervised clustering approach not only revealed the presence of eight antihypertensive drug related clusters including 10 different drugs, it also clustered metabolites of other drug types and endogenous urinary metabolites. Metformin, an antidiabetic, was annotated to a cluster containing a variety of precursor ions in the nodes. Indeed, the spectra shared the typical nitrogen-rich product ions of metformin and the urine extracts containing metformin were derived from four patients of whom three were reported to have diabetes, thereby identifying metformin in a male patient (aged 63) not reported to have diabetes. Many of the clustered nodes could be tentatively annotated as metformin conjugates with small organic acids. Metabolites of another drug-like cluster were found in three of the urine samples with the metformin metabolites, and could be annotated as a sulfonylurea type of drug, consistent with gliclazide based on fragment information also commonly prescribed to diabetes patients. Another drug-like cluster was annotated by GNPS with ranitidine, a histamine H2-receptor antagonist (inhibiting stomach acid production), with 20 separate metabolites present in one sample, indicating the extensive metabolism to which this drug is subject. Ranitidine is available in the UK both as prescription and over-the-counter formulations. No literature was found describing such extensive ranitidine metabolism. This finding reveals that common, over-the-counter drugs that might be in use for other medical reasons can also be identified and if applied to large studies on drug compliance and action, will offer an objective approach to seeking possible drug–drug interaction effects. Finally, two suspected drug metabolite clusters were annotated with MSI MI level 4 metabolites showing characteristic fragmentation patterns but without annotated core structure.

Altogether, a total of 165 different antihypertensive or other drug related metabolites were annotated in the urine extracts: 17 by spectral matching (MSI MI level 2) and 122 by classification based on the clustered fragmentation patterns (MSI MI level 3). This ultimately led to the detection of metabolite clusters of 13 different drugs in the studied urine extracts. All drug metabolites that were annotated in this study are described in Supplementary Table S3 with their theoretical masses, elemental formulas, annotation (with details on database or reference), MSI MI level, parent drug, and the drug class. A simplified Excel Table without annotation details is also available (Supplementary Table 3B). Although, in line with standard procedures, the urine spot tests are analysed in a blinded fashion, we can monitor that patient 61 was on losartan and bisoprolol, that patient 66 administered enalapril, bisoprolol, metformin, and likely gliclazide (a sulfonylurea class drug), and also took paracetamol, and that patient 91 was on losartan, perindopril, and atenolol, and also took paracetamol.

Molecular networking also clustered endogenous urinary metabolites based on their fragmentation patterns. Table 2 lists nine annotated clusters that include different biological compound classes including acylcarnitines (carnitine based), glutamine-related metabolites, and trigonelline related compounds. Most of these clusters were found using the combined fragmentation mode (first column in Table 2) with the betaine based cluster only found using the separate fragmentation mode (second column in Table 2). Trigonelline related compounds can be found in many plants and trigonelline has a vitamin B related structure; hence those metabolites could be either food-derived or endogenous break-down products of Vitamin B. In-depth metabolite annotation of all the clustered nodes of these nine clusters was outside the scope of this study; however, we did compare the masses detected in the acylcarnitine cluster with those previously annotated in a study using fragmentation data (van der Hooft et al. 2015); and substantial overlap between the masses was clear. The third column in Table 2 also showed that of four compound classes, at least one associated metabolite is present in all 26 urine extracts, illustrating their wide occurrence in humans. A series of glutamine containing compounds is one of those clusters and fragmentation spectra are consistent with a family of acylglutamines, i.e., glutamine-based analogues of acylcarnitines with different acyl chains. Very little literature exists discussing the existence or roles for acyl glutamines, and yet their abundance and diversity indicates they may represent a family of molecules with important metabolic functioning. Molecular networking can clearly assist in classifying urinary metabolites and aid in the annotation and identification of endogenous metabolites as well as xenobiotics.

**Table 2:** Table describing annotated clusters of non-drug metabolites, with ‘Compound class’ being the annotated core structure for each cluster, ‘Nodes in cluster’ being the number of nodes in each annotated cluster from the combined and separate fragmentation mode, respectively, and ‘MaxUniqueFileCount’ showing the occurrence of the most widely distributed nodes for each cluster

Compound class	Nodes in cluster combined fragmentation mode — POS	Nodes in cluster separate fragmentation mode — POS	MaxUniqueFileCount No. unique urine files (# nodes)
Carnitine based	52	52	26 (5), 25 (6)
Glutamine based	18	18	26 (3), 25 (2)
Trigonelline based	12	11	26 (1), 11(1)
Betaine based	−	11	12 (4), 8 (1) [POS only]
Steriod skeleton	2 + 2	12	11 (1), 8 (2)
Pyrriline-CO based	16	9	15 (1), 9 (3)
Pipecolic acid based	20	12	26 (4), 25 (2)
Lysine based	9	7	24 (1), 19 (1)
N containing oxygen rich substructure	10	10	2 (4), 1 (6)
Total	137	142	N/A

## Discussion

The combination of high-resolution untargeted mass spectrometry data-dependent fragmentation spectra and molecular networking has enabled the identification of a multitude of drugs and their metabolites in human urine samples. With over 5000 MS2 fragmentation scans obtained in each fragmentation measurement, and 26 urine fragmentation measurements, the number of MS2 spectra requiring processing and analysis counts in the tens of thousands. Advanced spectral analysis tools are needed to fully exploit the structural information present in fragmentation data and the molecular networking approach clearly offers a means to derive important information from such large and complex datasets.

Our approach was based on an existing HILIC chromatography based metabolomics platform (Creek et al. 2011), thereby focusing on polar and charged urinary metabolites. No single existing liquid chromatography platform can separate all drug metabolites efficiently in one run, and in our approach the majority of drug metabolites eluted between 3.5 and 4.5 min; however, a considerable number also elute in the period afterwards. Coupling HILIC-based liquid chromatography to Orbitrap high resolution spectrometry allows for the simultaneous detection of a wide range of polar urinary compounds in both positive and negative ionization modes. Our analysis focused on positive ionization mode which was compatible with most drugs, bearing several nitrogen atoms and thus easily ionizing in positive mode. Furthermore, with positive ionization mode generally resulting in more product ions per metabolite than negative ionization mode, the occurrence of drug related clusters was favoured in positive ionization mode.

Most reported LC–MS based drug methods are either focused on structural elucidation of one specific drug and its major pre-ascertained metabolites in biofluids or on the detection of multiple drugs through one drug related metabolite (usually the administrated drug or the active compound in case of pro-drugs). Our approach offers the ability to screen for many different types of drugs, irrespective of any expectation of their presence. Furthermore up to 20 different metabolites of a single drug were readily identified in the case of ranitidine which indicates how this approach can offer the means to initiate comprehensive studies into drug metabolism with relative ease.

It was encouraging to observe that many edges (connections between the cluster nodes) displayed typical mass differences of 176.032 (glucuronidation), 14.015 (methylation), and 16.000 Da (hydroxylation) that are commonly associated to drug (or xenobiotic) metabolism in man. Indeed, the described workflow is biased towards heavily metabolized core structures, since they will appear as distinct highly interlinked clusters in the molecular network. However, with more reference data becoming available in the future (i.e., increasing seed node annotation), the clusters of two or three drug metabolites may be detected more easily.

The structural identification of detected metabolites remains a bottleneck in untargeted metabolomics approaches (Creek et al. 2014; van der Hooft et al. 2013; Li et al. 2013; Roux et al. 2012; Dunn et al. 2013; Kind and Fiehn 2010; Wishart 2011). Generic fragmentation pathways for a number of antihypertensive and other drugs are available in the literature (Niessen 2011); however, high-resolution reference data of drug metabolites is still scarce and the comparison of fragmentation spectra across different platforms is not always straightforward. Moreover, the available structural information is often scattered across databases or tabulated within publications. Many databases generated by the Pharmaceutical industry are proprietary and thus not accessible. Mass spectral databases thus remain far from being a comprehensive representation of the contents of a given biological extract (Vinaixa et al. 2015; da Silva et al. 2015).

The current study shows that spectral clustering and matching enhances metabolite annotation and classification; however, extensive manual interpretation and validation remain essential for confident assessment of metabolite structures. For example, the drug clusters of the paracetamol mercapturates, enalapril and perindopril, amlodipine, the sulfonylurea class drug, and quinidine were annotated using amalgamated information from published literature values for drug product ions, MagMa (Fig. 3), or MzCloud spectral matching. Moreover, using the key fragments for each drug or drug family (see Supplementary Table S4), 45 additional drug metabolites were annotated that were not directly represented by a node within the molecular network. Our approach can map different classes of drugs and with the expected growth in spectral databases it certainly has the potential to group other xenobiotics and endogenous urinary metabolites as well. In fact, we could annotate a number of endogenous human metabolite clusters. For example, the acylcarnitine based cluster contained 52 nodes and of the masses of the 15 most widely distributed nodes (in >20 urine extracts present) all but 3 were indeed previously annotated as acylcarnitines (van der Hooft et al. 2015). The validation process also identified nodes that represented specific atomic isotopes (in chlorine-containing drugs like amlopidine) or adducts with co-eluting abundant metabolites like urea, which were removed from further analysis and annotation.

The amlodipine and ARB (sartan-based) drug clusters were analysed in more detail. For amlodipine, selected product ions could be matched to those reported from rat urine metabolites (Suchanova et al. 2006, 2008) alongside fragmentation data for two chemically synthesised oxidized amlodipine metabolites that we also observed in the human urine extracts (Suchanova et al. 2006). A study on 14C-labelled amlodipine metabolism in two humans (Beresford et al. 1988), revealed oxidized amlodipine metabolites that were excreted in the urine and an abundant COOH-metabolite of oxidized amlodipine, which matches well with our results. Our findings, which were derived with no preconception on which drugs or metabolites present in the samples, shows how the method can make important contributions to drug metabolism analysis without any need for bespoke reagents. Moreover, the study shows that the COOH-metabolite of oxidized amlodipine could be a better marker for amlodipine intake than the currently used amlodipine parent drug (Lawson et al. 2015). Losartan and hydroxylated losartan were found to be the most abundant drug metabolites present in the five urine samples containing metabolites related to this drug. Indeed, losartan was detected in plasma for up to 24 h after intake (Lo et al. 1995), indicating that this drug is excreted into the urine intact in most people. We found two closely eluting and related, but discernible isomers. One is likely to be the active EXP3174 metabolite (McCrea et al. 1999), and the other a ketone derivative (more analytical work would be required to confirm their structures). Critically we can identify differences between patients in the presence of these isomers, which indicates that the technique can be used to classify patients based on their ability to metabolise drugs. This in turn can then be linked to patient response data and offers the potential to predict patient response.

Our approach not only detected clusters of drug metabolites, but also biochemically relevant clusters of endogenous urinary compounds, as is illustrated by a number of different acyl amino acid families. Difference in levels of specific acyl amino acids has been directly linked to metabolic diseases (Chace 2009), and their enhanced annotation might facilitate future correlations between phenotypes and metabolites. Since it is also possible to identify other xenobiotics (e.g. non-antihypertensive drugs or even food related metabolites, note the impact of grapefruit juice on losartan pharmacokinetics (Zaidenstein et al. 2001; Bailey et al. 1998)), the potential to contribute to understanding drug metabolism and patient response may extend far beyond the simple identification of drugs and metabolites, but enable multidimensional correlation analysis offering predictive precision medicine. Furthermore, non-declared secondary health problems for which patients are taking drugs can be more easily spotted.

## Conclusions

Medication nonadherence is a common problem with up to 50 % of patients not adherent to long-term medications. However, drug adherence phenotype is not a simple trait and different tools are required to differentiate subtypes as this has implications on how nonadherence is treated (Marcum et al. 2013). There are limitations to the currently used methods of testing drug adherence and newer methods that can detect not only drug adherence agnostically but also reveal underlying physiology are essential. This study reveals the power of combining untargeted urinary metabolomics with molecular networking to guide the interpretation of large numbers of urine metabolite fragmentation spectra and identify, in an untargeted way, many drugs and metabolites associated with them. As a result, we report the key fragments we observed for each drug cluster based on spectral comparisons as well as an annotated table of all the validated metabolites in the clusters. In addition to offering utility in drug compliance testing (where parent drug or abundant metabolites can indicate whether patients have been taking the drugs) the approach also enables discrimination of metabolites in different people. If combined with large studies where treatment outcomes are also considered it may be possible to correlate certain metabolites with patient response to drug and thus offer novel tests to guide precision patient centred medicine. Moreover, the approach can also detect other drugs irrespective of prior knowledge of their presence which can also offer insights into drug–drug interactions. The expected increase in coverage of spectral databases and increasingly available in silico tools for metabolite annotation based on fragmentation data will aid in identification of drug metabolites not currently present in mass spectral libraries. Paracetamol-mercaptures were successfully annotated in our study with the aid of the in silico fragmenter MAGMa. Since endogenous metabolites are detected in the same datasets, the approach may detect roles for these in drug response and metabolism as well.

## Electronic supplementary material

Below is the link to the electronic supplementary material.

Supplementary material 1 (DOCX 1738 kb)

Supplementary material 1 (XLSX 19 kb)

Supplementary material 1 (DOCX 53 kb)

Supplementary material 1 (XLSX 21 kb)

Supplementary material 1 (CYS 3731 kb)
