# Supplementary material for: Urinary antihypertensive drug metabolite screening using molecular networking coupled to high-resolution mass spectrometry fragmentation
Source: Metabolomics. 2016 Jul 5;12:125. doi: 10.1007/s11306-016-1064-z (PMC4932139; doi:10.1007/s11306-016-1064-z)
Supplement: Supplementary file 1 — Supplementary material 1 (DOCX 1738 kb) [file 11306_2016_1064_MOESM1_ESM.docx]

**Supplementary material belonging to ‘Urinary antihypertensive drug metabolite screening using molecular networking coupled to high-resolution mass spectrometry fragmentation’**

**Supplementary Figures and Tables:**

**Section 1:**


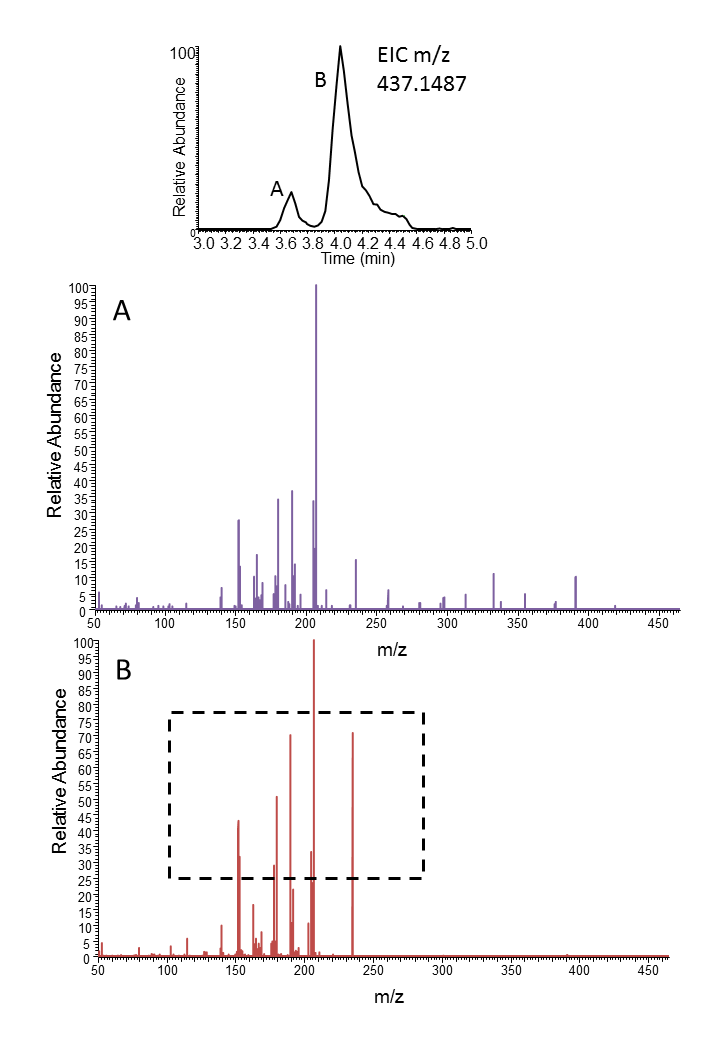


**Supporting Figure S1** The MS2 fragmentation spectra of two isomeric losartan related metabolites A and B with m/z 437.1487 [(M+H)^+^], with product ions differing in abundance indicated with a dashed box in the lower spectrum.

**Supporting Table S1** Separate Excel file with all information on which fragmentation experiments were conducted for which urine extracts and the recorded patient information consisting of: i) general information like age, gender, ii) records indicating which drug classes they were taking at the time of the study, and iii) serum measurement values. The abbreviations for the different antihypertensive drug classes are as follows:

ACE I angiotensin converting enzyme inhibitors (drugs ending on -pril)

ARB angiotensin type II receptor blockers (drugs ending on -sartan)

Diuretic promoting production of urine (drugs often ending on -zide)

Statin low-density lipoprotein blood level lowering drugs (drugs ending on -statin)

β-blocker beta-adrenergic blocking agent (drugs often ending on -olol)

α-blocker adrenergic inhibitors

Ca Antag Calcium-channel blockers (two types - dihydropyridines (drugs ending on -ipine)

and non-dihydropyridines)

NSAID non-steroidal anti-inflammatory drugs (i.e., iboprufen)

Nitrate Anti-anginal drugs

**Supporting Table S2** All molecular networking parameters and their values used to generate the molecular networks used in the manuscript.

|  | Parameter | Value |
| --- | --- | --- |
| 1 | PAIRS_MIN_COSINE | 0.55 |
| 2 | ANALOG_SEARCH | 1 |
| 3 | tolerance.PM_tolerance | 0.25 |
| 4 | tolerance.Ion_tolerance | 0.005 |
| 5 | MIN_MATCHED_PEAKS | 2 |
| 6 | TOPK | 10 |
| 7 | CLUSTER_MIN_SIZE | 2 |
| 8 | MAXIMUM_COMPONENT_SIZE | 120/100* |
| 9 | MIN_PEAK_INT | 500.0 |
| 10 | FILTER_STDDEV_PEAK_INT | 2.0 |
| 11 | RUN_MSCLUSTER | On |
| 12 | FILTER_PRECURSOR_WINDOW | 0 |
| 13 | FILTER_LIBRARY | 1 |
| 14 | WINDOW_FILTER | 0 |
| 15 | SCORE_THRESHOLD | 0.6 |
| 16 | MIN_MATCHED_PEAKS_SEARCH | 4 |
| 17 | MAX_SHIFT_MASS | 100.0 |

*120 for combined fragmentation mode files, 100 for separate fragmentation mode files. These values were determined by starting at 80 and increase in steps of 20 till the largest network was smaller than the maximum component size.

**Supporting Table S3** Separate word file with all annotated drug related metabolites. For each metabolite, the theoretical mass ([M+H ]^+^), elemental formula ([M+H ]^+^), retention time, annotation (metabolite description and further information if available), metabolomics standards initiative metabolite identification (MSI MI) level, the parent drug of the drug metabolite, and its drug class are recorded. The annotations contain references to metabolites found in literature and the MSI MI level contains information to which spectral database a spectral match was found. Masses in italic were annotated in the network, their network annotation are also given in the table.

**Supporting Table S3B** Separate Excel file of Supporting Table S3 with shortened (simplified) metabolite annotations only containing the metabolite descriptions.

**Supporting Table S4** Key fragments annotated to core structures of annotated drugs in this study, with ‘Parent drug’ representing the annotated parent drug, and ‘Characteristic product ions’ the key fragments of each drug cluster, with the theoretical masses and elemental formulas.

| Parent drug | Characteristic product ions  Theoretical m/z of product ions ([M+H]^+^) and the elemental formula | |  |
| --- | --- | --- | --- |
| Losartan / Irbesartan | **207.0917,**  **190.0651,**  **180.0808** | **C14H11N2,**  **C14H8N,**  **C13H10N** | |
| Verapamil | **165.0910,**  **150.0675,**  **260.1645** | **C10H13O2,**  **C9H10O2,**  **C16H12NO2** | |
| Clopidogrel | **125.0153,**  **141.0102,**  **113.0153** | **C7H6Cl,**  **C7H6OCl,**  **C6H6Cl** | |
| Enalapril | **117.0699.**  **160.1121** | **C9H9,**  **C11H14N** | |
| Perindopril | **98.0964,**  **170.1176,**  **172.1332,**  **124.1121** | **C6H12N,**  **C9H16NO2,**  **C9H18NO2,**  **C8H14N** | |
| Atenolol/  Bisoprolol | **145.0648,**  **74.0600,**  **116.1070** | **C10H9O,**  **C3H8NO,**  **C6H14NO** | |
| Paracetamol (PC) | **152.0706,**  **110.0600,**  **65.0386** | **C8H10NO2,**  **C6H8NO,**  **C5H5** | |
| Amlodipine | **286.0265,**  **258.0316,**  **230.0367,**  **167.0730** | **C15H9NO3Cl,**  **C14H9NO2Cl,**  **C13H9NOCl,**  **C12H9N** | |
| PC-mercapturate | **140.0165,**  **182.0270,**  **96.0444,**  **166.0321** | **C6H6NOS,**  **C8H8NO2S,**  **C5H6NO,**  **C8H8NOS** | |
| Ranitidine | **102.0372,**  **125.0056,**  **130.0559,**  **170.0634** | **C4H8NS,**  **C6H5SO,**  **C5H10N2S,**  **C8H12NOS** | |
| Metformin | **113.0822,**  **60.0556,**  **71.0604** | **C4H9N4,**  **CH6N3,**  **C3H7N2** | |
| Sulfonylurea drug class (possibly gliclazide) | **91.0542,**  **143.1179,**  **155.0161** | **C7H7,**  **C7H15N2O,**  **C7H7SO2** | |
| Quinidine | **160.0757,**  **82.0651,**  **172.0757** | **C10H10NO,**  **C5H8N,**  **C11H10NO** | |

**Supporting Table S5** All drugs detected in the study, with ‘Parent Drug’ representing the annotated parent drug, ‘Metabolites in clusters’ being the number of unique metabolites found in the molecular networks of the combined and separate fragmentation mode files, ‘Total metabolites found in data’ being the total number of metabolites annotated based on the clusters and characteristic product ions, ‘MaxUniqueFileCount’ being the number of unique urine extracts in which one or more drug metabolites was found, and ‘Urine extracts that contain one or more drug metabolites’ specifying which urine extracts contained one or more drug metabolites of each cluster.

| Parent Drug | Metabolites in clusters | Total metabolites  found in data | MaxUniqueFileCount  (from combined fragmentation files) | Urine extracts that contain one or more drug metabolites |
| --- | --- | --- | --- | --- |
| **Losartan** | **6** | **10** | **5** | **37, 61, 87, 91, 96** |
| **Irbesartan** | **8** | **12** | **1** | **73** |
| **Verapamil** | **13** | **21** | **2** | **05, 23** |
| **Clopidogrel** | **9** | **21** | **2** | **37, 44** |
| **Enalapril** | **3** | **3** | **4** | **05, 19, 66, 67** |
| **Perindopril** | **6** | **6** | **2** | **76, 91** |
| **Atenolol** | **3** | **4** | **13** | **19, 22, 44, 60, 64, 73, 76, 84, 87, 88, 91, 95** |
| **Bisoprolol** | **2** | **2** | **4** | **25, 61, 66, 86, 101** |
| **Paracetamol (PC)** | **2** | **2** | **12** | **05, 14, 37, 44, 64, 66, 73, 84, 91, 95, 96, 101** |
| **Amlodipine** | **5** | **5** | **7** | **19, 20, 67, 76, 84, 88, 90** |
| **PC-mercapturate** | **11** | **11** | **11** | **05, 14, 37, 44, 64, 66, 73, 74, 84, 96, 101** |
| **Ranitidine** | **10** | **20** | **1** | **14** |
| **Metformin** | **14** | **20** | **4** | **23, 66, 84, 90** |
| **Sulfonylurea drug class (possibly gliclazide)** | **5** | **6** | **3** | **23, 66, 90** |
| **Quinidine** | **8** | **10** | **1** | **67** |
| **Unknown-C9H11NOSbased** | **7** | **7** | **4** | **05, 37, 44, 73** |
| **Unknown-C15H17N2O7Sbased** | **5** | **5** | **1** | **19** |
| **Validated** | **120** | **165** | **N/A** | **N/A** |

**Section 2: All drug related clusters from both combined and separate fragmentation mode molecular networks as present in the networks**


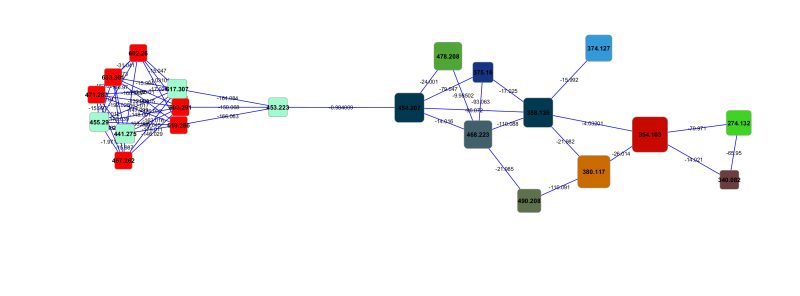

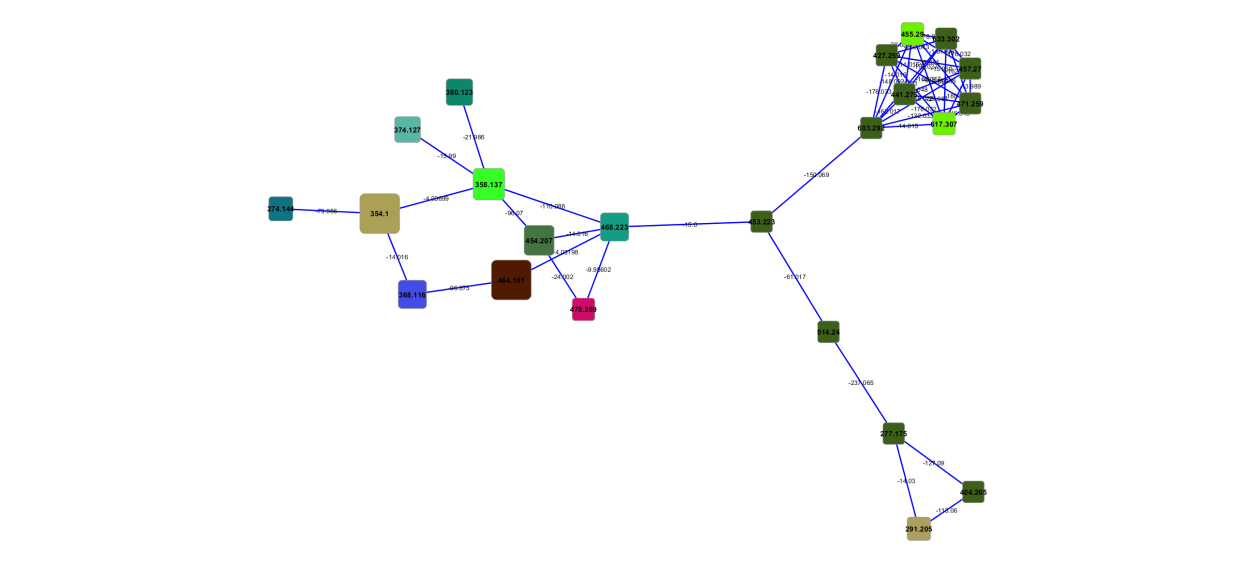


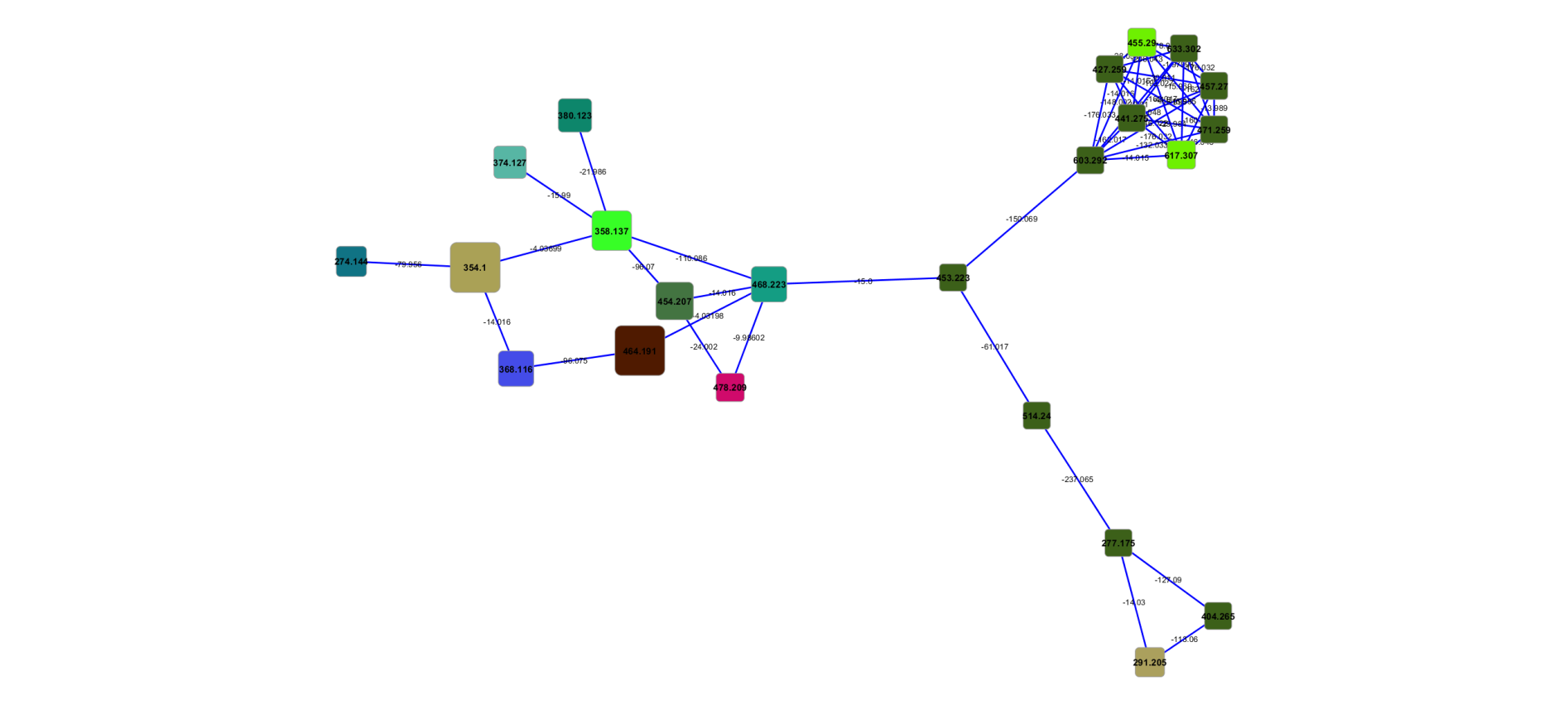


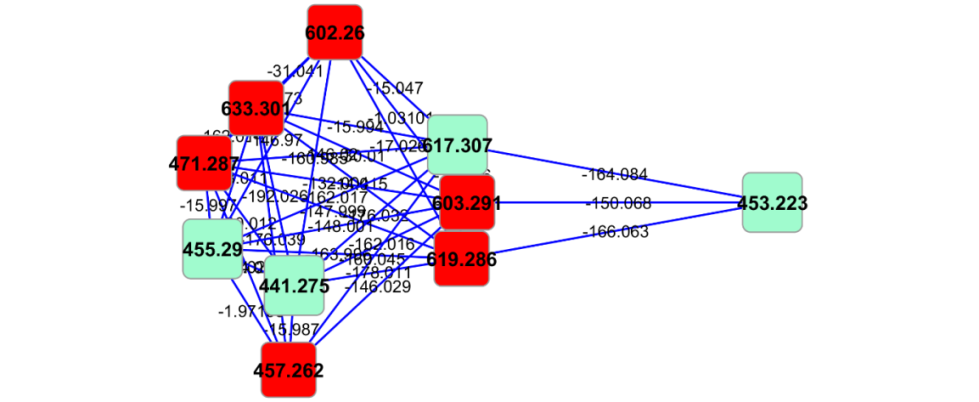


**Supporting Figure S2** Top: complete verapamil clusters as found in molecular networks (combined fragmentation mode left, separate fragmentation mode right), and Bottom: verapamil clusters in molecular networks (combined fragmentation mode left, separate fragmentation mode right) displayed for more detailed views of the nodes; in total, 2 unique urine extracts contained one or more verapamil related metabolites.


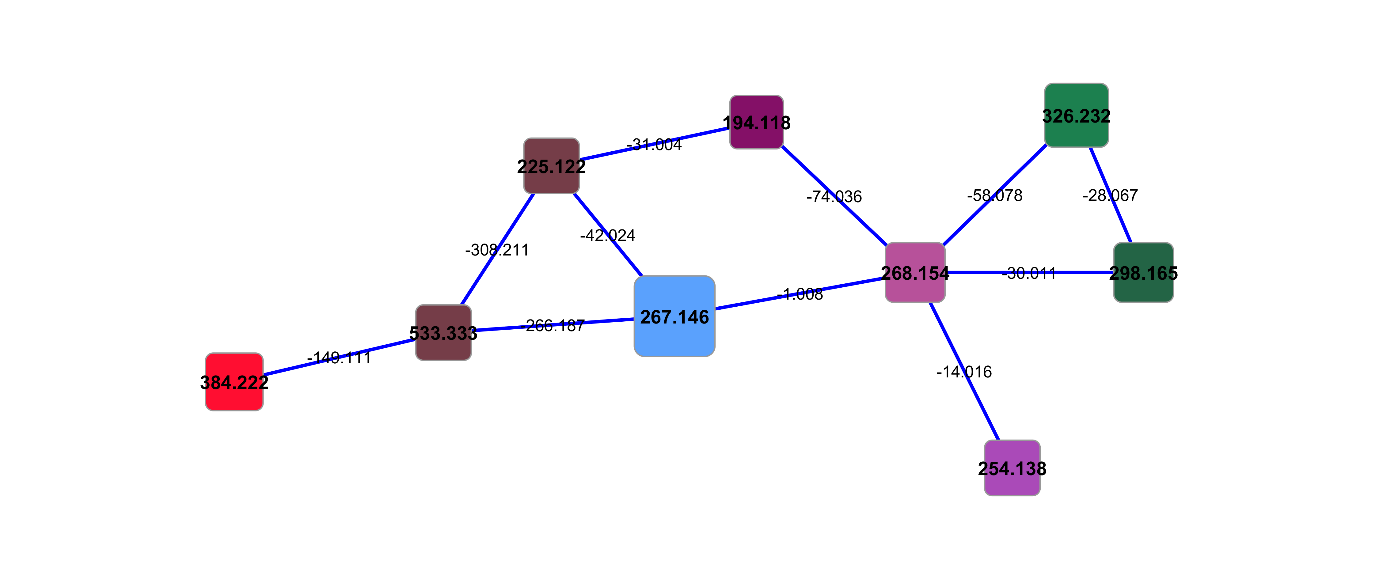

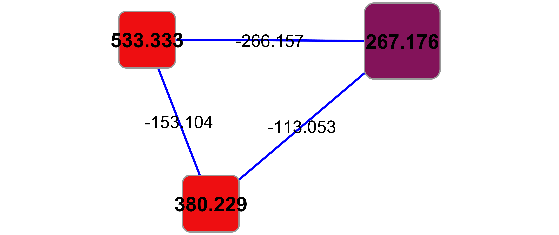


**Supporting Figure S3** Atenolol and bisoprolol clusters as found in molecular networks (combined fragmentation mode left, separate fragmentation mode right); in total, 13 unique urine extracts contained one or more atenolol related metabolites, and 4 unique urine extracts contained one or more bisoprolol related metabolites.


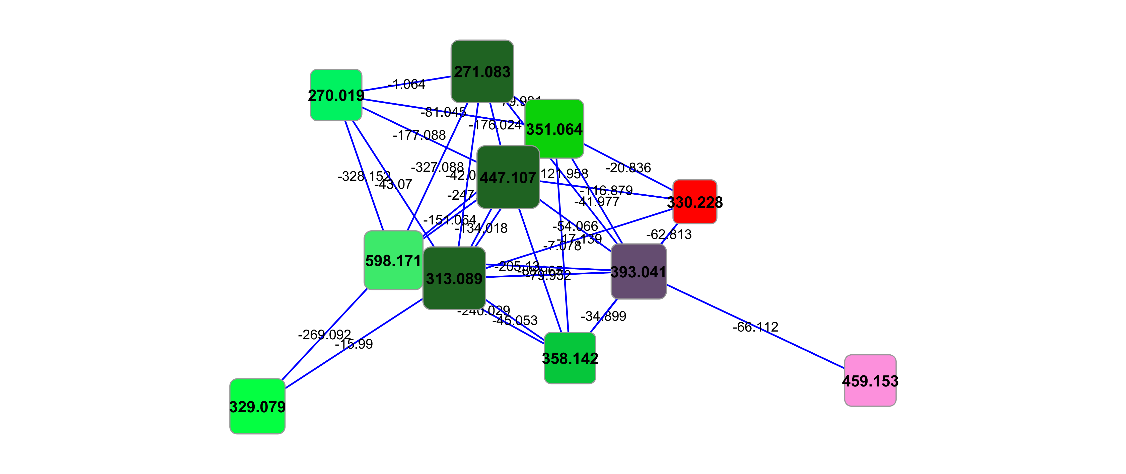

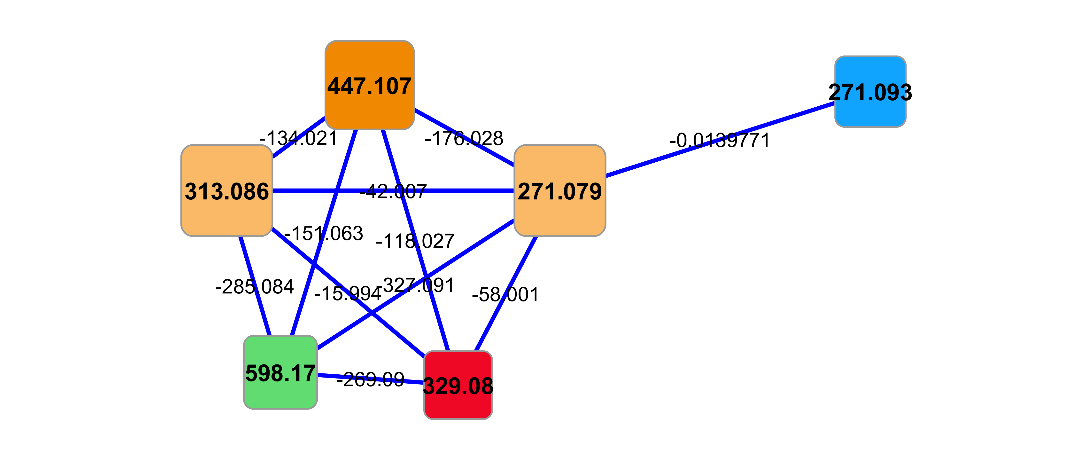


**Supporting Figure S4** Paracetamol mercapturate clusters as found in molecular networks (combined fragmentation mode left, separate fragmentation mode right); 13 unique urine extracts contained one or more paracetamol mercapturate related metabolites.


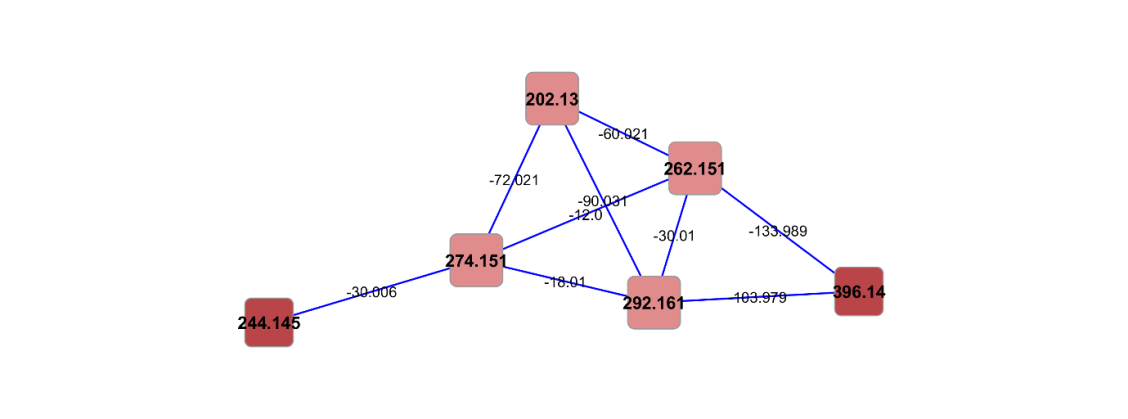

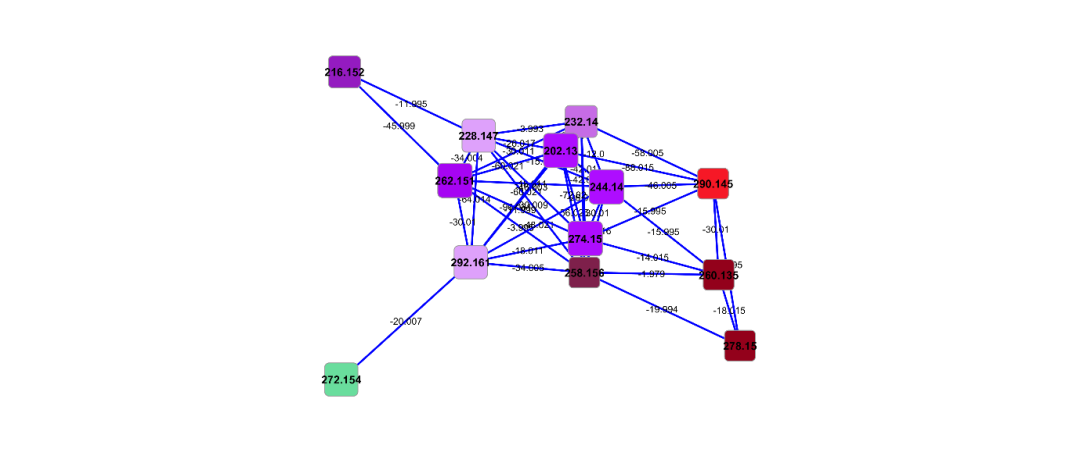


**Supporting Figure S5** Metformin clusters as found in molecular networks (combined fragmentation mode left, separate fragmentation mode right); 4 unique urine extracts contained one or more metformin related metabolites.


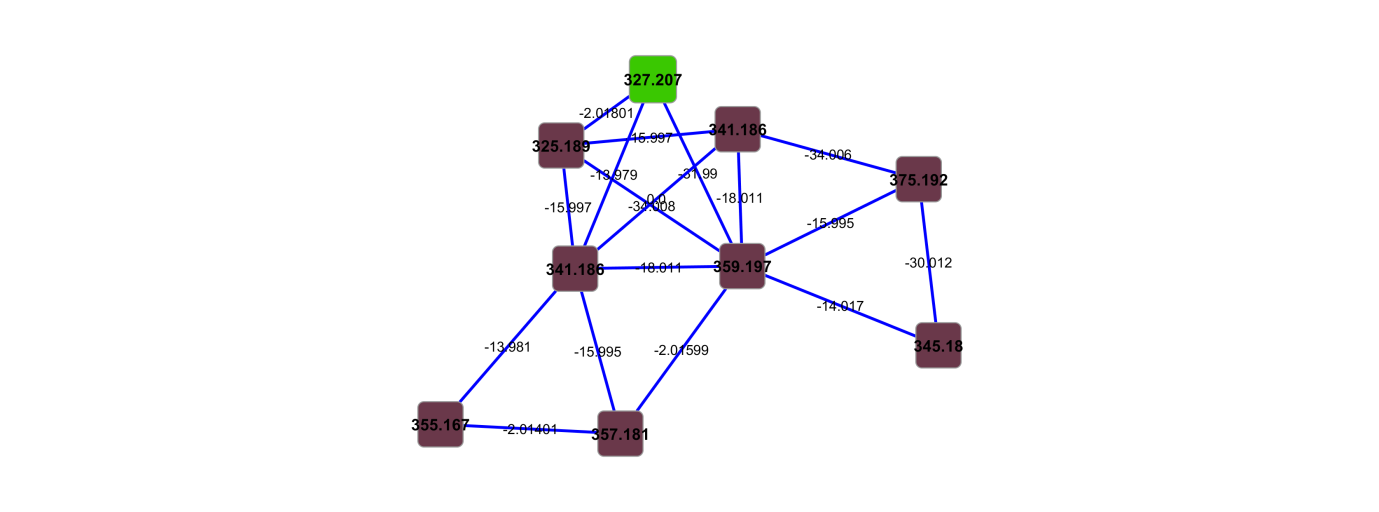


**Supporting Figure S6** Quinidine cluster as found in molecular network (combined fragmentation mode);

1 unique urine extract contained quinidine related metabolites.


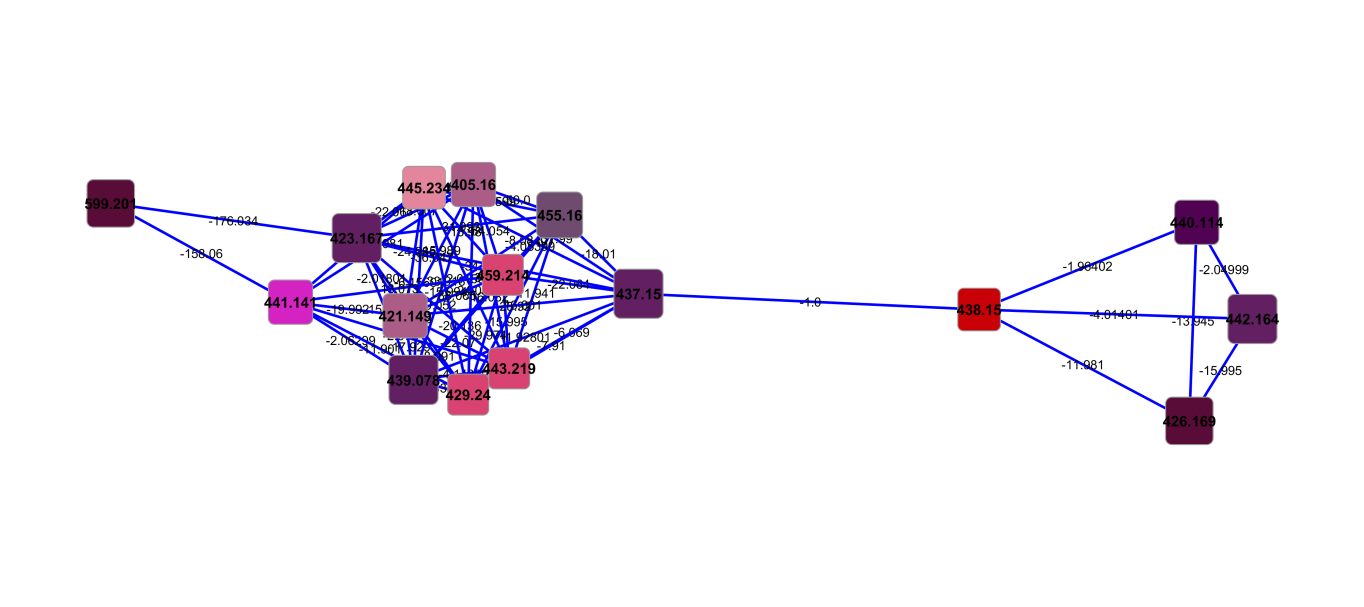

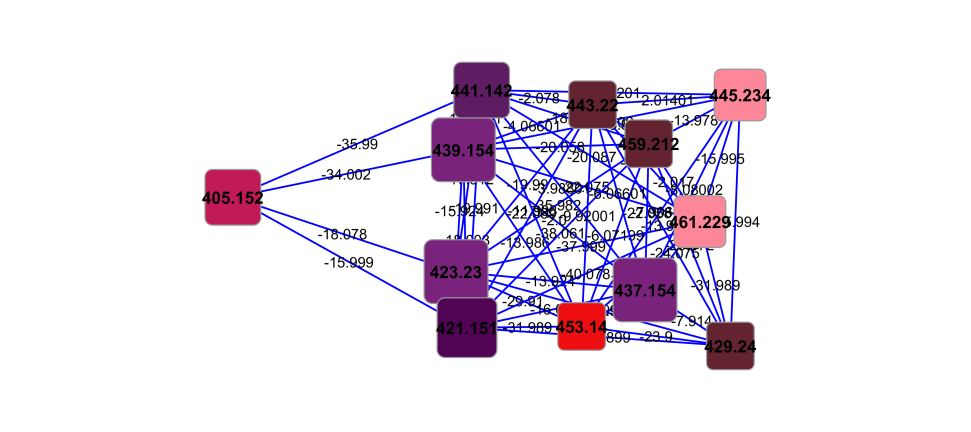


**Supporting Figure S7** Sartan (Irbesartan and Losartan) clusters as found in molecular networks (combined fragmentation mode top, separate fragmentation mode bottom); 1 unique urine extract contained irbesartan related metabolites, and 5 unique urine extracts contained one or more losartan related metabolites.


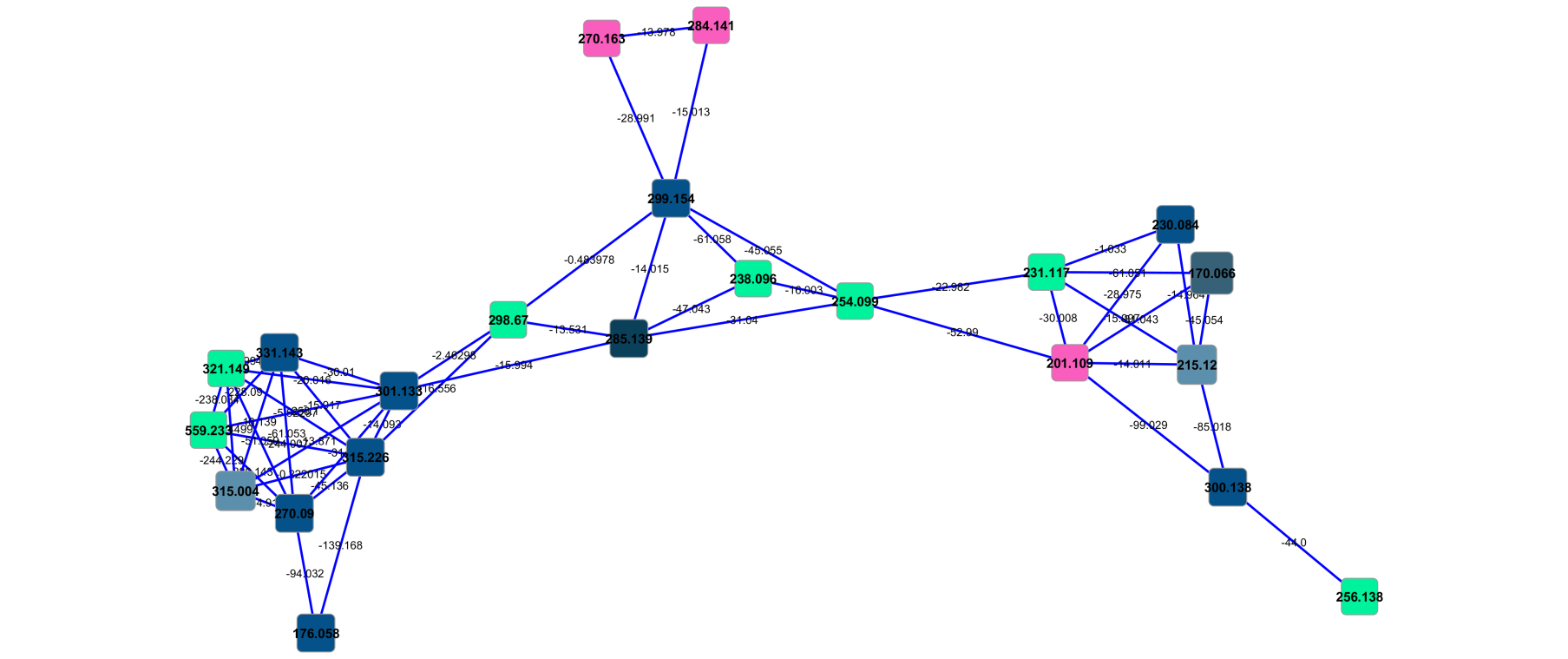


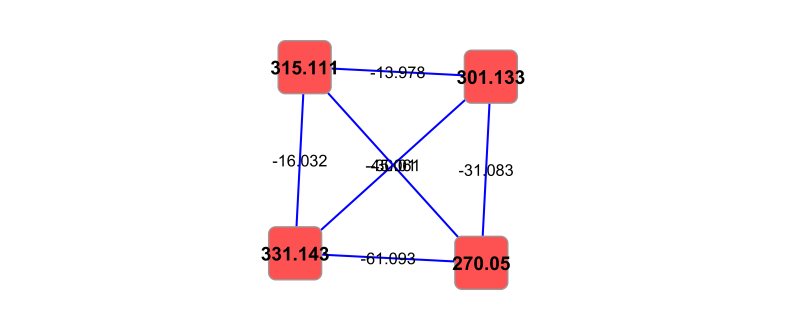


**Supporting Figure S8** Ranitidine clusters as found in molecular networks (combined fragmentation mode top, separate fragmentation mode bottom); 1 unique urine extract contained ranitidine related metabolites.


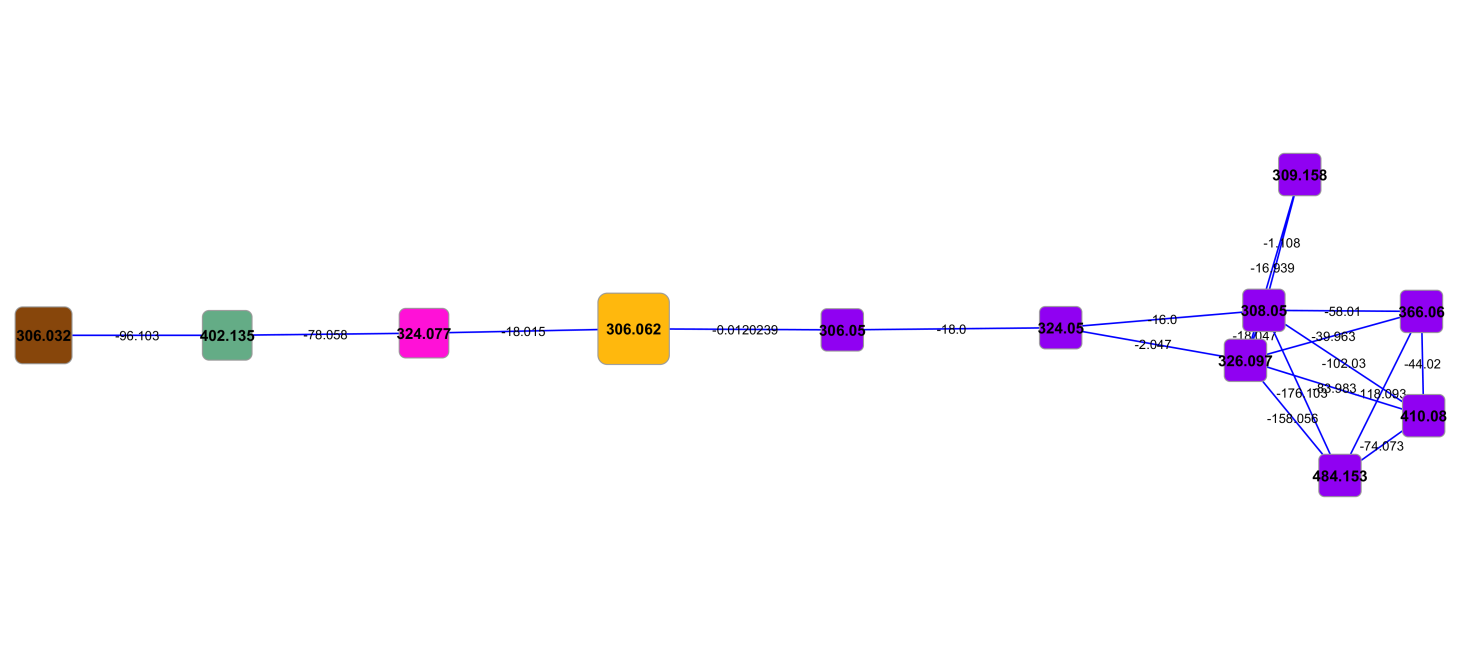

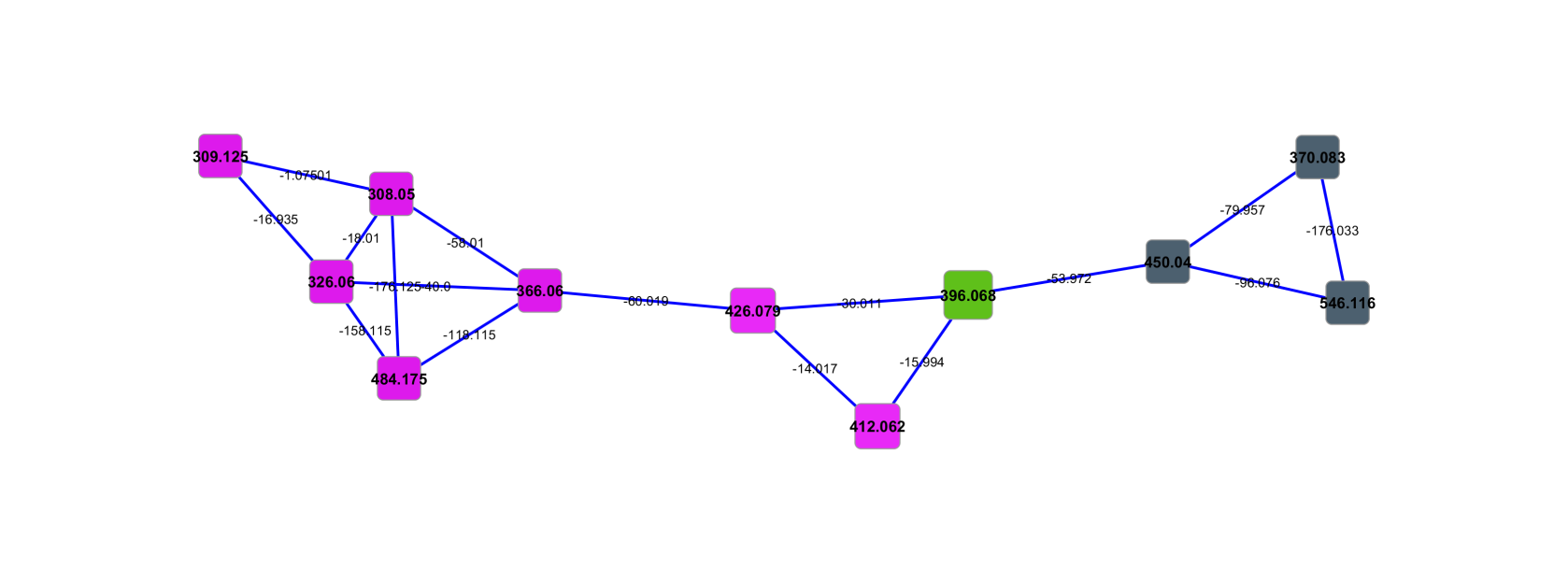


**Supporting Figure S9** Clopidogrel clusters as found in molecular networks (combined fragmentation mode top, separate fragmentation mode bottom); 2 unique urine extract contained one or more clopidogrel related metabolites.

Unknown drug C15H17N2O7S-based metabolites and an unknown drug C9H11NOS-based metabolite are linked to clopidogrel metabolites in combined fragmentation mode.


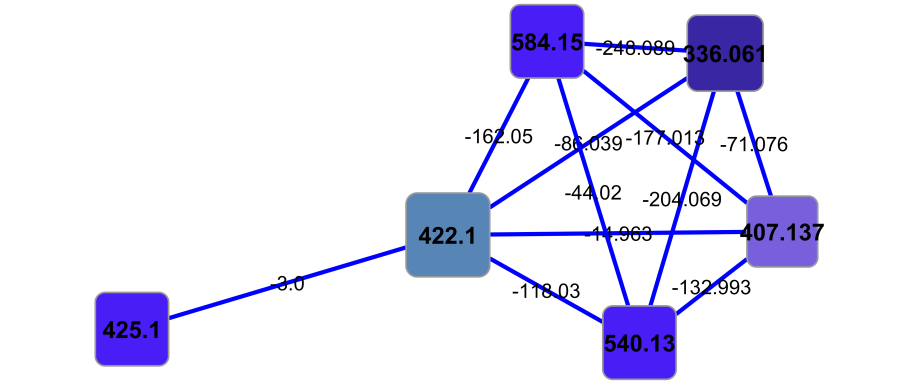


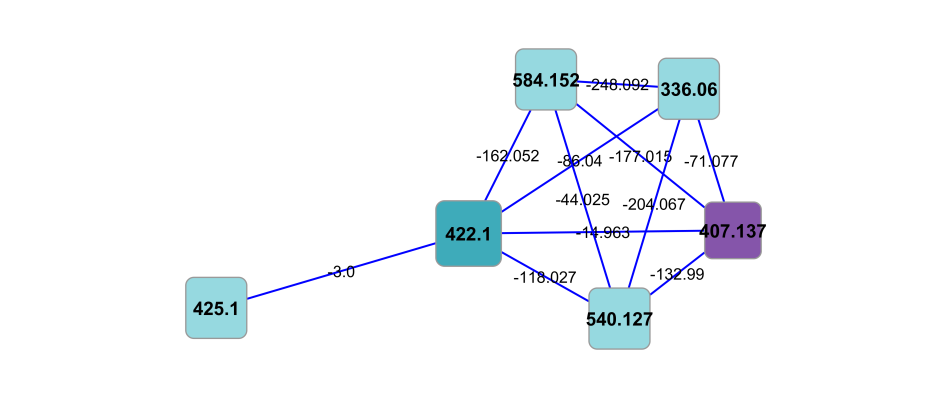


**Supporting Figure S10** Amlodipine clusters as found in molecular networks (combined fragmentation mode top, separate fragmentation mode bottom); 7 unique urine extract contained one or more amlodipine related metabolites.


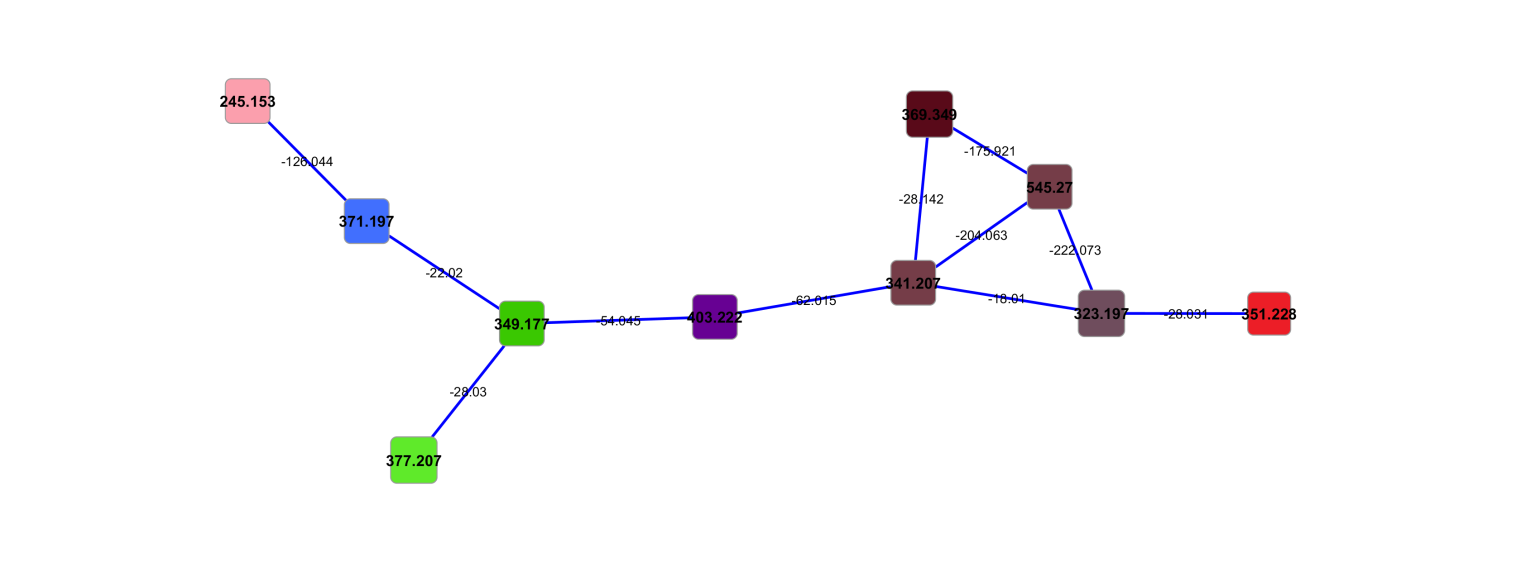


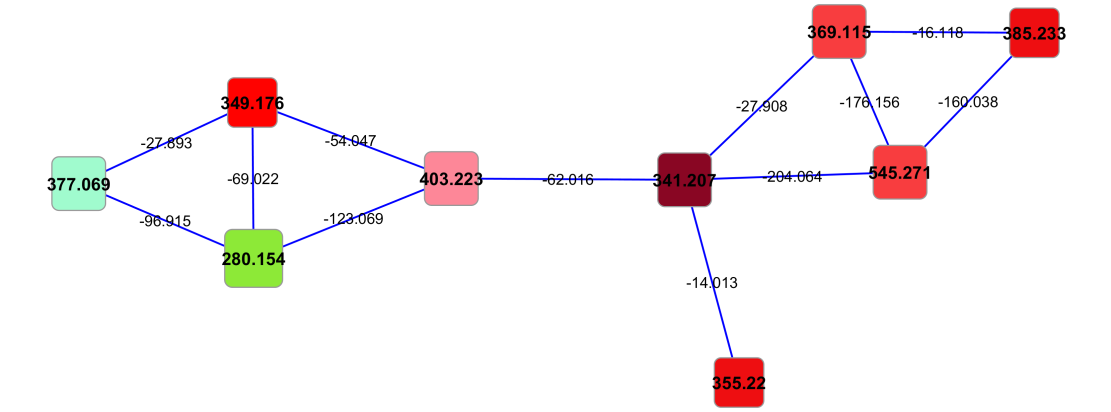


**Supporting Figure S11** Prilats based clusters as found in molecular networks (combined fragmentation mode top, separate fragmentation mode bottom); 4 unique urine extract contained one or more enalapril related metabolites, and 2 unique urine extracts contained one or more perindopril related metabolites.


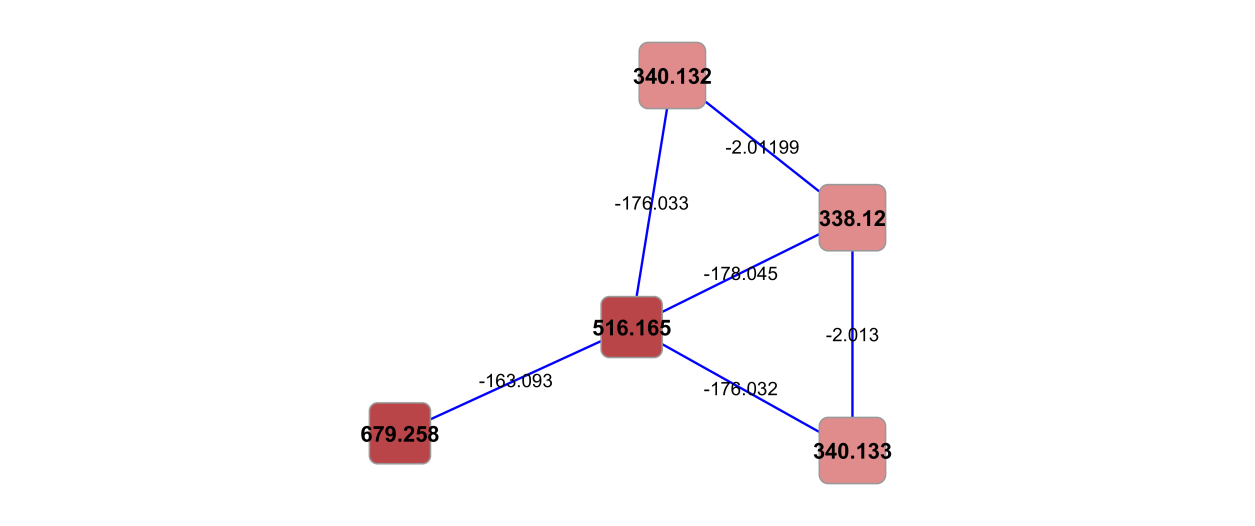


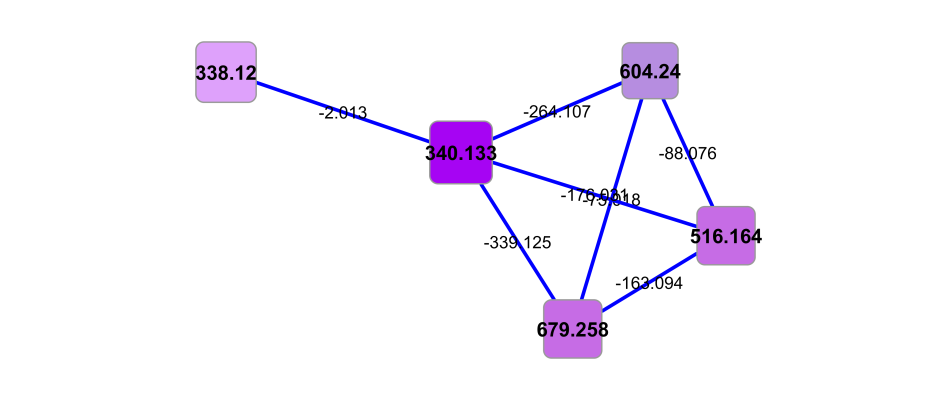


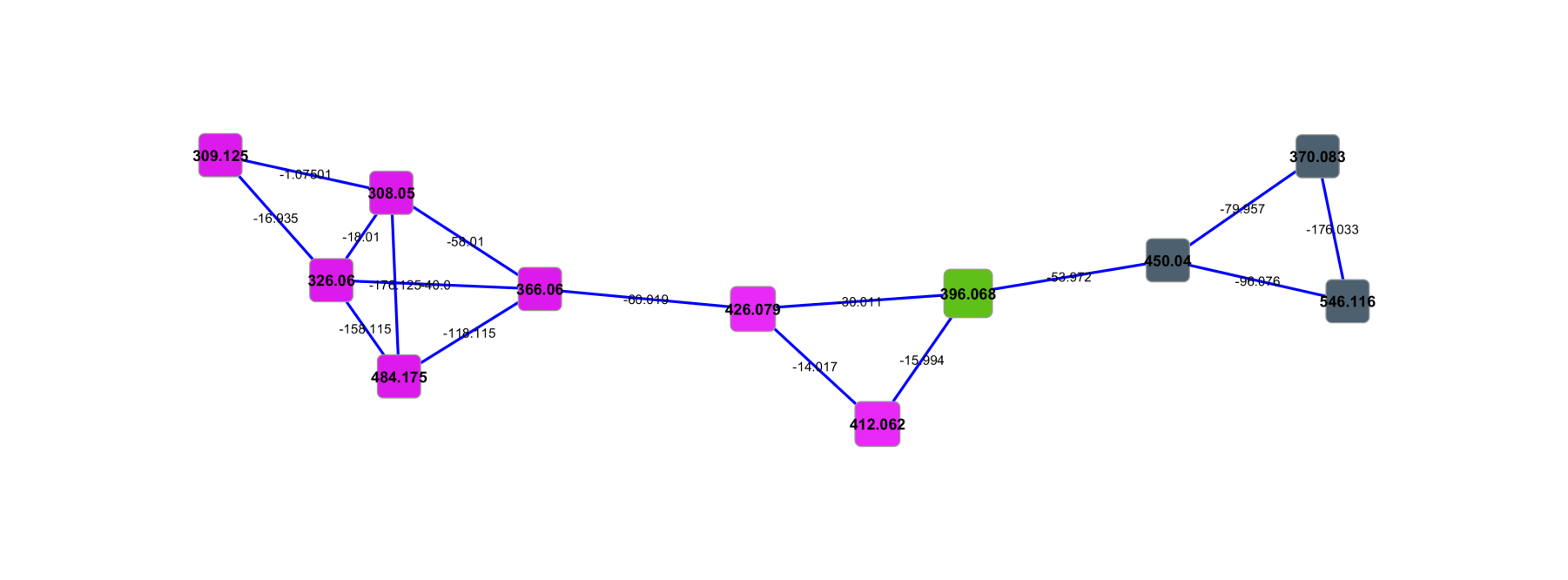
**Supporting Figure S12** Sulfonylurea drug clusters as found in molecular networks (combined fragmentation mode left, separate fragmentation mode right); 3 unique urine extract contained one or more sulfonylurea drug related metabolites.


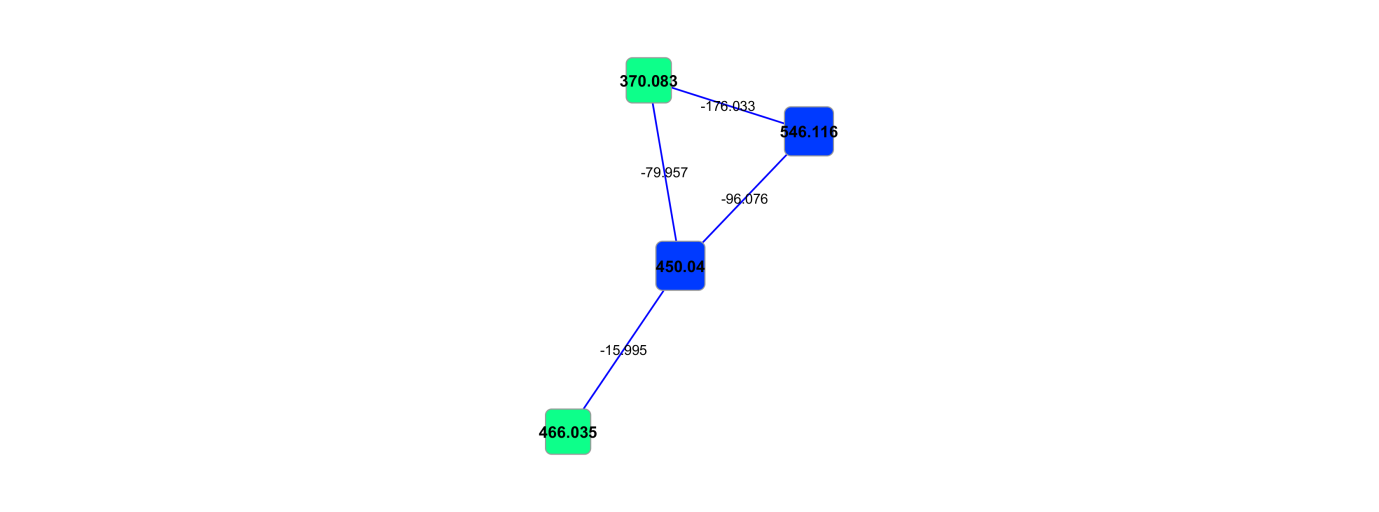


**Supporting Figure S13** Unknown C15H17N2O7S-based drug clusters as found in molecular networks (combined fragmentation mode top, separate fragmentation mode bottom); 1 unique urine extract contained unknown drug C15H17N2O7S-based metabolites.

Clopidogrel metabolites are linked to unknown drug C15H17N2O7S-based metabolites and an unknown drug C9H11NOS-based metabolite in combined fragmentation mode.


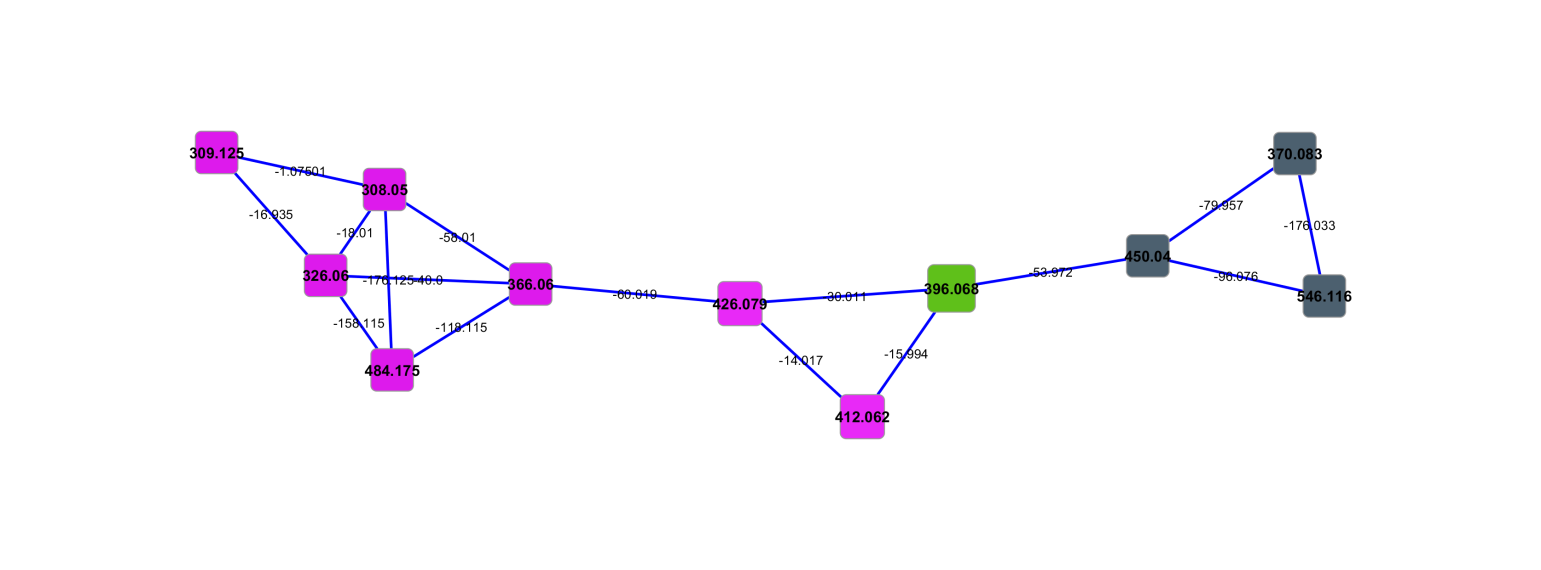


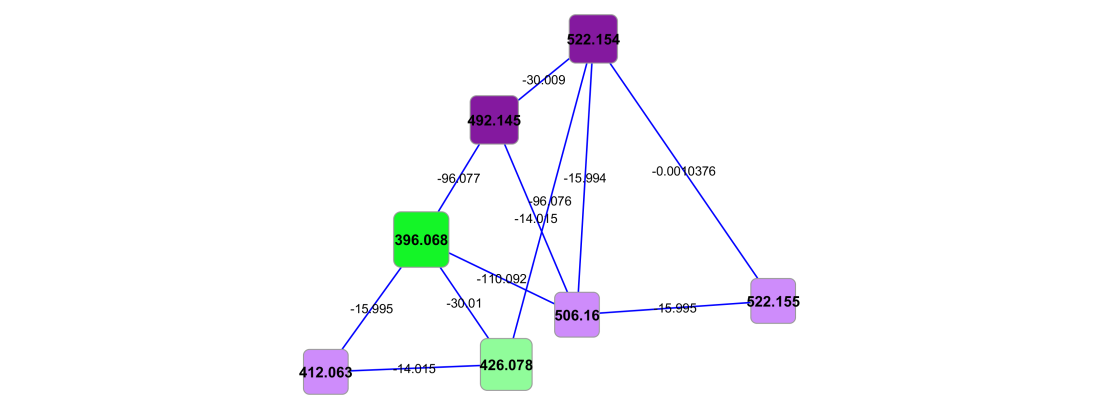


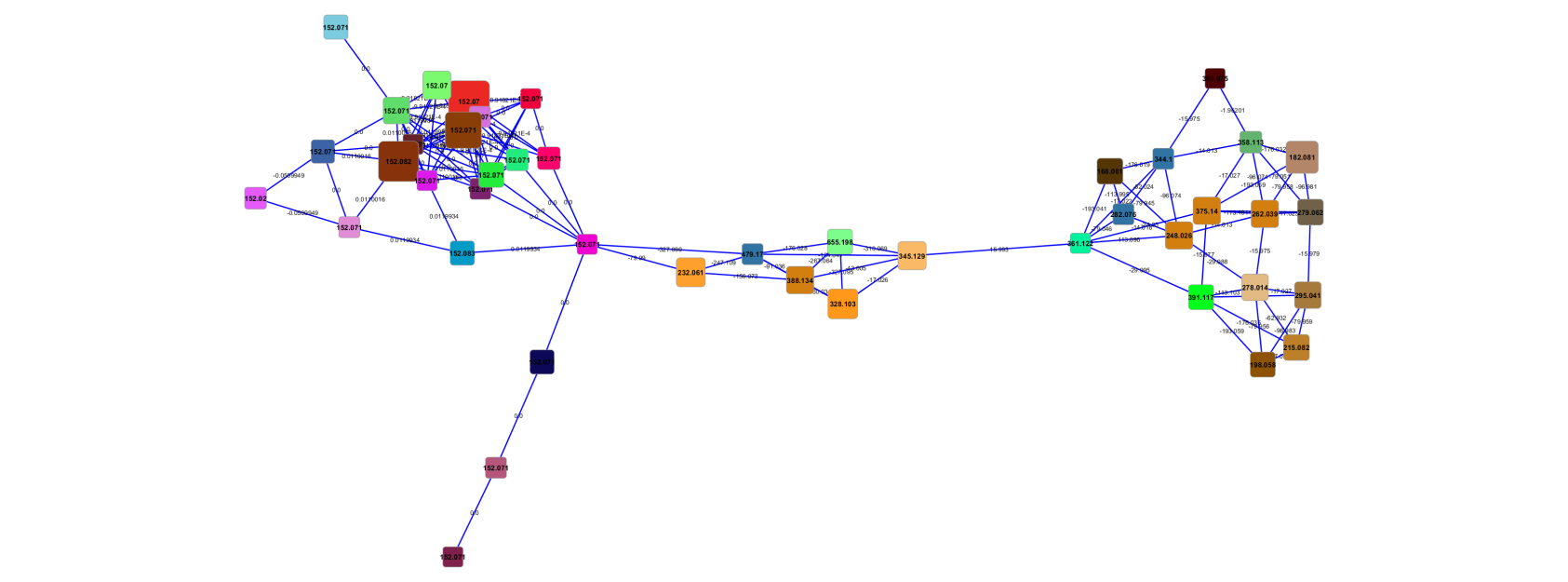
**Supporting Figure S14** Unknown C9H11NOS-based drug clusters as found in molecular networks (combined fragmentation mode left, separate fragmentation mode right); 4 unique urine extract contained one or more unknown drug C9H11NOS-based metabolites.


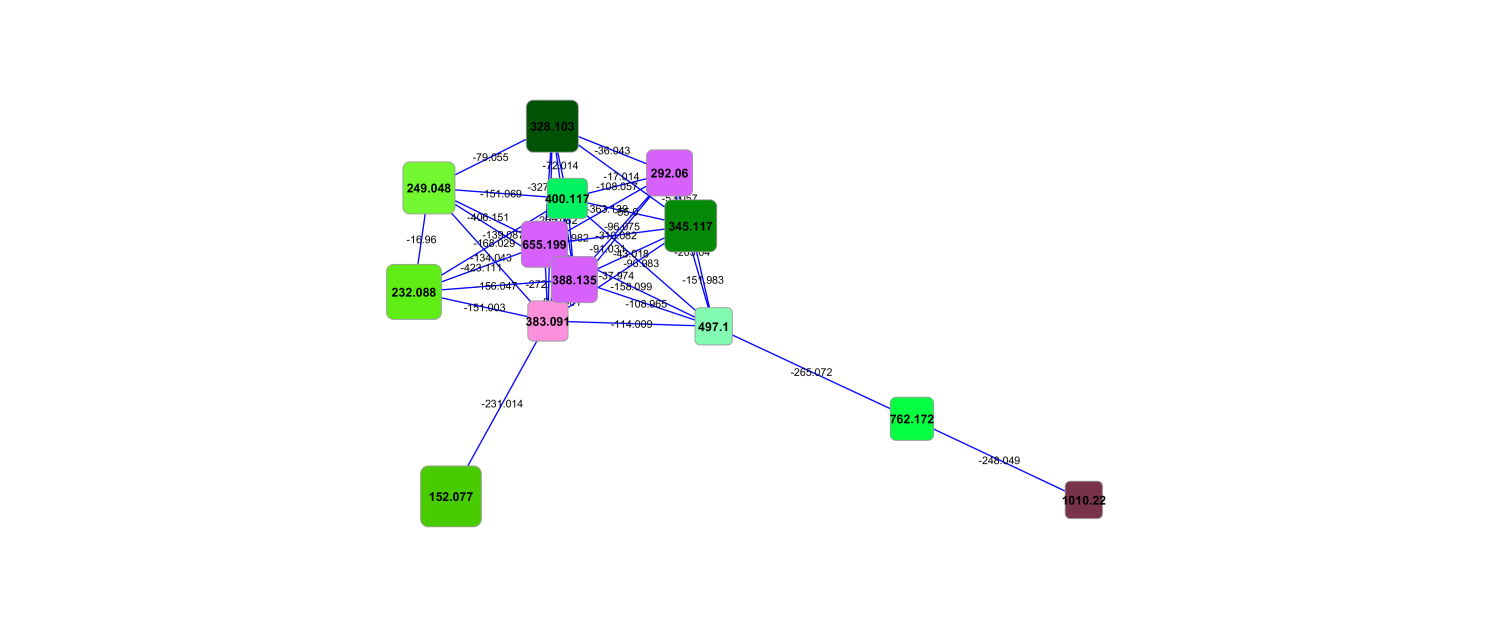

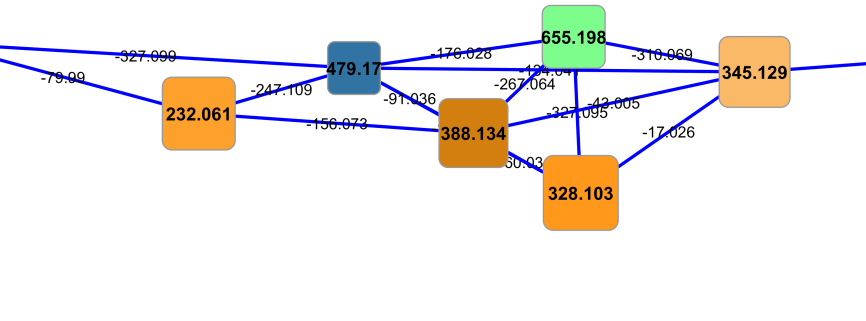


**Supporting Figure S15** Paracetamol based drug clusters as found in molecular networks (combined fragmentation mode top with zoom on two paracetamol drug metabolites, and separate fragmentation mode bottom); 12 unique urine extract contained one or more paracetamol related metabolites.
