# Supplementary material for: Urinary antihypertensive drug metabolite screening using molecular networking coupled to high-resolution mass spectrometry fragmentation
Source: Metabolomics. 2016 Jul 5;12:125. doi: 10.1007/s11306-016-1064-z (PMC4932139; doi:10.1007/s11306-016-1064-z)
Supplement: Supplementary file 3 — Supplementary material 1 (DOCX 53 kb) [file 11306_2016_1064_MOESM3_ESM.docx]

| Mass ([M+H]^+^)  m/z | Elemental formula  ([M+H]) | Retention  Time (min) | Annotation | MSI MI level | Parent drug | Drug class |
| --- | --- | --- | --- | --- | --- | --- |
| 349.1758 | C18H25N2O5 | 4.6 | Enalaprilat | 2 (MzCloud) | Enalapril | ACE inhibitor |
| 377.2071 | C20H29N2O5 | 3.9 | Enalapril | 3 | Enalapril | ACE inhibitor |
| 371.1965 | C21H27N2O4 | 3.9 | Enalapril related mtb | 3 | Enalapril | ACE inhibitor |
| 341.2071 | C17H29N2O5 | 4.8 | Perondropilat | 3 | Perondropril | ACE inhibitor |
| 369.2384 | C19H33N2O5 | 3.8 | Perondropril | 3 | Perondropril | ACE inhibitor |
| 351.2278 | C19H31N2O4 | 4.0 | Perondropril related mtb | 3 | Perondropril | ACE inhibitor |
| 355.2227 | C18H31N2O5 | 4.0 | Perondropril related mtb | 3 | Perondropril | ACE inhibitor |
| 385.2333 | C19H33N2O6 | 4.0 | Perondropril - hydroxylated | 3 | Perondropril | ACE inhibitor |
| 545.2705 | C25H41N2O11 | 3.9 | Perondropril - glucuronidated | 3 | Perondropril | ACE inhibitor |
| *308.0507* | C15H15NO2ClS | 4.1 | Clopidogrel-COOH mtb  *In network: Clopidogrel carboxylic acid*  *Massbank* | 2 (MzCloud) | Clopidogrel | ADP binding |
| 322.0663 | C16H17NO2ClS | 4.0 | Clopidogrel | 2 (MzCloud) | Clopidogrel | ADP binding |
| 326.0612 | C15H17NO3ClS | 4.6 | Clopidogrel 2-oxo like mtb I | 3 | Clopidogrel | ADP binding |
| 326.0612 | C15H17NO3ClS | 6.5 | Clopidogrel 2-oxo like mtb II | 3 | Clopidogrel | ADP binding |
| 484.0827 | C21H23NO8ClS | 4.1 | Clopidogrel-COOH mtb - Glc | 3 | Clopidogrel | ADP binding |
| 366.0561 | C17H17NO4ClS | 3.7 | Clopidogrel + CO2 mtb | 3 | Clopidogrel | ADP binding |
| 354.0561 | C16H17NO4ClS | 3.8 | Clopidogrel 2-oxo with modified acetate? | 3 | Clopidogrel | ADP binding |
| 324.0997 | C16H19NO4Cl | 4.0 | Clopidogrel active thiol mtb minus thiol (SH) group? | 3 | Clopidogrel | ADP binding |
| 324.0456 | C15H15NO3ClS | 4.2 | Clopidogrel 2-oxo mtb | 3 | Clopidogrel | ADP binding |
| 426.0773 | C19H21NO6ClS | 3.7 | Clopidogrel-COOH mtb with succinate conjugate? | 3 | Clopidogrel | ADP binding |
| 410.0823 | C19H21NO5ClS | 3.8 | Clopidogrel-COOH mtb with erythrose conjugate? | 3 | Clopidogrel | ADP binding |
| 340.0405 | C15H15NO4ClS | 4.4 | Clopidogrel-COOH mtb with thiol group? | 3 | Clopidogrel | ADP binding |
| 340.0946 | C16H19NO5Cl | 4.0 | Clopidogrel-O-COOH mtb with open sulfated ring minus thiol (SH) group? Isomer I | 3 | Clopidogrel | ADP binding |
| 340.0946 | C16H19NO5Cl | 4.3 | Clopidogrel-O-COOH mtb with open sulfated ring minus thiol (SH) group? Isomer II | 3 | Clopidogrel | ADP binding |
| 342.1103 | C16H21NO5Cl | 4.1 | Clopidogrel-O-COOH mtb with open sulfated ring minus thiol (SH) group, oxidized? | 3 | Clopidogrel | ADP binding |
| 530.0882 | C22H25NO10ClS | 4.1 | Clopidogrel-O-COOH 2-oxo mtb glucuronidated | 3 | Clopidogrel | ADP binding |
| 514.0399 | C22H25NO9ClS | 4.2 | Clopidogrel oxidized (-2H) Glc | 3 | Clopidogrel | ADP binding |
| 546.1195 | C23H29NO10ClS | 4.0 | Clopidogrel - Clopidogrel-O-COOH mtb with open sulfated ring and methylated and Glc | 3 | Clopidogrel | ADP binding |
| 516.1090 | C22H27NO9ClS | 4.2 | Clopidogrel Glc | 3 | Clopidogrel | ADP binding |
| 552.0497* | ? | 4.2 | Possible Copidogel mtb – typical fragments in MS2 | 4 | Clopidogrel | ADP binding |
| 370.0874 | C17H21NO4ClS | 3.8 | Clopidogrel-O-COOH mtb with open sulfated ring and methylated | 3 | Clopidogrel | ADP binding |
| *429.2397* | C25H29N6O | 3.6 | Irbesartan  *In network: Irbesartan (fda_library)*  *M9 in Chando et al., 1998* | 2 (MzCloud) | Irbesartan | Ang II Ant |
| 427.2241 | C25H27N6O | 3.6 | Irbesartan oxidized (-2H) | 3 | Irbesartan | Ang II Ant |
| 459.2139 | C25H27N6O3 | 3.9 | Irbesartan COOH or keton hydroxyl mtb - isomer I  *M2 or M3 in Chando et al., 1998* | 3 | Irbesartan | Ang II Ant |
| 459.2139 | C25H27N6O3 | 4.5 | Irbesartan COOH or keton hydroxyl mtb - isomer II  *M2 or M3 in Chando et al., 1998* | 3 | Irbesartan | Ang II Ant |
| 443.2190 | C25H27N6O2 | 3.6 | Irbesartan keton mtb (most likely on the spirocyclopentane ring)  *M6 in Chando et al., 1998* | 2 | Irbesartan | Ang II Ant |
| 445.2347 | C25H29N6O2 | 3.7 | Irbesartan hydroxylated  *M4, M5, or M7 in Chando et al., 1998* | 3 | Irbesartan | Ang II Ant |
| 461.2296 | C25H29N6O3 | 4.0 | Irbesartan dihydroxylated  *M1 in Chando et al., 1998* | 2 | Irbesartan | Ang II Ant |
| 477.2248 | C25H29N6O4 | 4.1 | Irbesartan trihydroxylated | 3 | Irbesartan | Ang II Ant |
| 549.2269 | C24H31N5O6 | 4.2 | Irbesartan unknown mtb | 3 | Irbesartan | Ang II Ant |
| 479.2401 | C25H31N6O4 | 4.4 | Irbesartan oxidized / hydroxylated mtb | 3 | Irbesartan | Ang II Ant |
| 605.2718 | C31H37N6O7 | 3.8 | Irbesartan Glc (most likely a tetrazole N2-beta-glucuronide conjugate)  *M8 in Chando et al., 1998* | 3 | Irbesartan | Ang II Ant |
| 621.2667 | C31H37N6O8 | 4.0 | Irbesartan hydroxylated and glucuronidated | 3 | Irbesartan | Ang II Ant |
| *437.1487* | C22H22N6O2Cl | 3.7 | Losartan COOH or keton hydroxyl mtb - isomer I  *In network: valsartan*  *(fda_library)* | 3 | Losartan | Ang II Ant |
| 437.1487 | C22H22N6O2Cl | 4.0 | Losartan COOH or keton hydroxyl mtb - isomer II (most abundant isomer, likely to be COOH metabolite – which is the active mtb  *EXP3174 in Schmidt and Schieffer, 2003.* | 3 | Losartan | Ang II Ant |
| *439.1644* | C22H24N6O2Cl | 3.8 | Losartan hydroxylated  *In network: valsartan*  *(fda_library)*  *M2, M5, or M6, in Schmidt and Schieffer, 2003.* | 3 | Losartan | Ang II Ant |
| 423.1695 | C22H24N6OCl | 3.6 | Losartan | 2 (MassBank) | Losartan | Ang II Ant |
| 599.2016 | C28H32N6O7Cl | 3.8 | Losartan glucuronide  *M3 or M4 in Schmidt and Schieffer, 2003.* | 3 | Losartan | Ang II Ant |
| 421.1538 | C22H22N6OCl | 3.7 | Losartan oxidized (-2H)  *EXP3179 in Schmidt and Schieffer, 2003.* | 3 | Losartan | Ang II Ant |
| 405.1589 | C22H22N6Cl | 3.6 | Losartan reduced (-O) | 3 | Losartan | Ang II Ant |
| 455.1593 | C22H24N6O3Cl | 4.0 | Losartan dihydroxylated – one OH group on aromatic ring | 3 | Losartan | Ang II Ant |
| 453.1436 | C22H22N6O3Cl | 4.8 | Losartan oxidized (-2H) dihydroxylated isomer I | 3 | Losartan | Ang II Ant |
| 453.1436 | C22H22N6O3Cl | 6.9 | Losartan oxidized (-2H) dihydroxylated isomer II | 3 | Losartan | Ang II Ant |
| 455.2904 | C27H39N2O4 | 4.1 | Verapamil | 2 (MzCloud) | Verapamil | Ca channel blocker |
| *441.2748* | C26H37N2O4 | 5.9 | Norverapamil  *In network: Verapamil*  *Massbank* | 2 (MzCloud) | Verapamil | Ca channel blocker |
| 427.2591 | C25H35N2O4 | 6.8 | Desmethylnorverapamil | 3 | Verapamil | Ca channel blocker |
| 457.2697 | C26H37N2O5 | 6.8 | Norverapamil hydroxylated | 3 | Verapamil | Ca channel blocker |
| 617.3069 | C32H45N2O10 | 4.4 | Norverapamil Glc Isomer I | 3 | Verapamil | Ca channel blocker |
| 617.3069 | C32H45N2O10 | 6.7 | Norverapamil Glc Isomer II | 3 | Verapamil | Ca channel blocker |
| 471.2854 | C27H39N2O5 | 4.2 | Verapamil hydroxylated | 3 | Verapamil | Ca channel blocker |
| 603.2912 | C31H43N2O10 | 4.8 | Desmethyl-Norverapamil Glc Isomer I | 3 | Verapamil | Ca channel blocker |
| 603.2912 | C31H43N2O10 | 6.8 | Desmethyl-Norverapamil Glc Isomer II | 3 | Verapamil | Ca channel blocker |
| 453.2231 | C22H33N2O8 | 8.1 | Norverapamil fragment C-N-C cleavage Glc | 3 | Verapamil | Ca channel blocker |
| 514.2395 | C23H36N3O10 | 4.2 | Norverapamil C-N-C cleavage fragment related mtb | 3 | Verapamil | Ca channel blocker |
| *277.1910* | C16H25N2O2 | 9.0 | Norverapamil fragment (resulting from C-N-C cleavage) Metabolite VI in Eichelbaum, 1979.  *In network: Verapamil metabolite D617*  *Massbank* | 3 | Verapamil | Ca channel blocker |
| *291.2067* | C17H27N2O2 | 8.0 | Verapamil fragment (resulting from C-N-C cleavage) Metabolite V in Eichelbaum, 1979.  *In network: Verapamil metabolite D617*  *Massbank* | 3 | Verapamil | Ca channel blocker |
| 410.2649 | C21H36N3O5 | 8.0 | Verapamil C-N-C cleavage fragment conjugate (or adduct) with amino acid | 3 | Verapamil | Ca channel blocker |
| 528.2552 | C24H38N3O10 | 4.0 | Verapamil C-N-C cleavage fragment related mtb | 3 | Verapamil | Ca channel blocker |
| 335.1965 | C18H27N2O4 | 4.0 | Verapamil C-N-C cleavage fragment carboxylated? | 3 | Verapamil | Ca channel blocker |
| 633.3018 | C32H45N2O11 | 4.0 | Norverapamil hydroxylated Glc Isomer I | 3 | Verapamil | Ca channel blocker |
| 633.3018 | C32H45N2O11 | 4.7 | Norverapamil hydroxylated Glc Isomer II | 3 | Verapamil | Ca channel blocker |
| 647.3174 | C33H47N2O11 | 4.5 | Verapamil hydroxylated Glc | 3 | Verapamil | Ca channel blocker |
| 507.2159 | C25H35N2O7S | 4.0 | Desmethylnorverapamil sulfate | 3 | Verapamil | Ca channel blocker |
| 321.1809 | C17H25N2O2 | 4.2 | Norverapamil C-N-C cleavage fragment carboxylated? | 3 | Verapamil | Ca channel blocker |
| *267.1703* | C14H23N2O3 | 11.3 | Atenolol  *In network: Atenolol*  *Massbank* | 2 (MzCloud) | Atenolol | Beta blocker |
| 268.1543 | C14H22NO4 | 8.8 | Atenolol-COOH (-NH2 + OH) | 3 | Atenolol | Beta blocker |
| 254.1387 | C13H20NO4 | 9.0 | Atenolol-COOH minus CH2 (methylgroup in alkyl chain) | 3 | Atenolol | Beta blocker |
| 298.1649 | C15H24NO5 | 8.4 | Bisoprolol related mtb | 3 | Bisoprolol | Beta blocker |
| *326.2326* | C18H32NO4 | 6.8 | Bisoprolol  *In network: Propanolol*  *Massbank* | 2 (MzCloud) | Bisoprolol | Beta blocker |
| 225.1234 | C11H17N2O3 | 11.4 | Atenolol desisopropyl | 2 (MoNa) | Atenolol | Beta blocker |
| 422.1001 | C20H21NO7Cl | 3.7 | Amlodipine oxidized with[-CH2-NH2] replaced by [-COOH]  *D1 in Suchanova et al., 2006*  *H-VII in Beresford et al., 1988* | 3 | Amlodipine | Ca channel blocker |
| 407.1368 | C20H24N2O5Cl | 6.8 | Amlodipine oxidized (-2H), likely to form a pyridine ring)  *D3 in Suchanova et al., 2006*  *H-IX in Beresford et al., 1988* | 3 | Amlodipine | Ca channel blocker |
| 336.0633 | C16H15NO5Cl | 4.0 | Amlodipine related mtb [-CH2CH2NH2 and – C2H4] | 3 | Amlodipine | Ca channel blocker |
| 584.1529 | C26H31NO12Cl | 4.0 | Amlodipine oxidized C20H23NO6Cl mtb - Glc | 4 | Amlodipine | Ca channel blocker |
| 540.1267 | C24H27NO11Cl | 4.0 | Amlodipine oxidized [–CH2CH2NH2] – Glc  *GLC of H-VI, in Beresford et al., 1988* | 3 | Amlodipine | Ca channel blocker |
| *315.1485* | C13H23N4O3S | 6.8 | Ranitidine  *In network: Ranitidine N-oxide*  *Massbank* | 2 (MzCloud) | Ranitidine | Histamine H2 Ant |
| *301.1329* | C12H21N4O3S | 9.6 | Desmethylranitidine  *In network: Ranitidine N-oxide*  *Massbank* | 3 | Ranitidine | Histamine H2 Ant |
| *331.1435* | C13H23N4O4S | 7.4 | Ranitidine N-oxide  *In network: Ranitidine*  *Massbank* | 2 (MzCloud) | Ranitidine | Histamine H2 Ant |
| 299.1536 | C13H23N4O2S | 8.4 | Ranitidine –O at NO2 group – likely followed by rearrangement - isomer I | 3 | Ranitidine | Histamine H2 Ant |
| 299.1536 | C13H23N4O2S | 8.9 | Ranitidine –O at NO2 group – likely followed by rearrangement - isomer II | 3 | Ranitidine | Histamine H2 Ant |
| 299.1536 | C13H23N4O2S | 11.4 | Ranitidine –O at NO2 group – likely followed by rearrangement - isomer III | 3 | Ranitidine | Histamine H2 Ant |
| 299.1536 | C13H23N4O2S | 11.8 | Ranitidine –O at NO2 group – likely followed by rearrangement - isomer IV | 3 | Ranitidine | Histamine H2 Ant |
| 387.1683 | C14H25N7O4S | 7.3 | Ranitidine related mtb (+CH2N3O?!) | 4 | Ranitidine | Histamine H2 Ant |
| 559.2335* | ? | 5.0 | Ranitidine related mtb | 4 | Ranitidine | Histamine H2 Ant |
| 559.2335* | ? | 7.2 | Ranitidine related mtb | 4 | Ranitidine | Histamine H2 Ant |
| 321.1485 | C27H46N8O6S2 | 7.8 | Ranitidine dimer like mtb isomer I [M+2H]^+^ | 4 | Ranitidine | Histamine H2 Ant |
| 321.1485 | C27H46N8O6S2 | 8.9 | Ranitidine dimer like mtb isomer II [M+2H]^+^ | 4 | Ranitidine | Histamine H2 Ant |
| 298.6705* | ? | 13.0 | Ranitidine related mtb [M+2H]^+^ | 4 | Ranitidine | Histamine H2 Ant |
| 238.0965 | C19H32N4O8S | 11.8 | Ranitidine N-glucuronide isomer I [M+2H]^+^ | 3 | Ranitidine | Histamine H2 Ant |
| 238.0965 | C19H32N4O8S | 12.3 | Ranitidine N-glucuronide isomer II [M+2H]^+^ | 3 | Ranitidine | Histamine H2 Ant |
| 286.1220 | C12H20N3O3S | 10.3 | Ranitidine –NH-CH3 group cleavage | 3 | Ranitidine | Histamine H2 Ant |
| 300.1376 | C13H22N3O3S | 7.0 | Ranitidine related mtb (–NH) isomer I | 4 | Ranitidine | Histamine H2 Ant |
| 300.1376 | C13H22N3O3S | 7.6 | Ranitidine related mtb (–NH) isomer II | 4 | Ranitidine | Histamine H2 Ant |
| 457.1751 | C19H29N4O7S | 7.5 | Ranitidine C6H8O5 conjugate, possibly alpha-ketoadipate | 3 | Ranitidine | Histamine H2 Ant |
| 617.2123 | C25H37N4O12S | 8.1 | Ranitidine related mtb | 4 | Ranitidine | Histamine H2 Ant |
| 274.1510 | C10H20N5O4 | 10.0 | Metformin C6H8O4 conjugate isomer I  ([hydroxy-adipate -H2O]?) | 3 | Metformin | Antidiabetic |
| 274.1510 | C10H20N5O4 | 15.7 | Metformin C6H8O4 conjugate isomer II  ([hydroxy-adipate -H2O]?) | 3 | Metformin | Antidiabetic |
| 274.1510 | C10H20N5O4 | 17.9 | Metformin C6H8O4 conjugate isomer III  ([hydroxy-adipate -H2O]?) | 3 | Metformin | Antidiabetic |
| 292.1615 | C10H22N5O5 | 15.6 | Metformin C6H10O5 conjugate ([hexose -H2O]) | 3 | Metformin | Antidiabetic |
| 262.1509 | C9H22N5O4 | 15.7 | Metformin C5H8O4 conjugate  ([pentose –H2O]?) | 3 | Metformin | Antidiabetic |
| 244.1404 | C9H18N5O3 | 15.9 | Metformin C5H6O3 conjugate ([glutarate –H2O]?) | 3 | Metformin | Antidiabetic |
| 130.1087 | C4H12N5 | 22.8* | Metformin | 2 (MzCloud) | Metformin | Antidiabetic |
| 258.1561 | C10H20N5O3 | 17.5 | Metformin C6H8O3 conjugate [(adipate –H2O)?] | 3 | Metformin | Antidiabetic |
| 260.1353 | C9H18N5O4 | 16.6 | Metformin C5H6O4 conjugate [(hydroxyglutarate –H2O)?] | 3 | Metformin | Antidiabetic |
| 278.1459 | C9H20N5O5 | 16.7 | Metformin C5H8O5 conjugate [(pentonate (sugary acid) –H2O)?] | 3 | Metformin | Antidiabetic |
| *216.1455* | C8H18N5O2 | 15.4 | Metformin C4H6O2 conjugate [(hydroxybutarate –H2O)?]  Isomer I  *In network: Metformin*  *(Massbank)* | 3 | Metformin | Antidiabetic |
| 216.1455 | C8H18N5O2 | 16.5 | Metformin C4H6O2 conjugate [(hydroxybutarate –H2O)?]  Isomer II | 3 | Metformin | Antidiabetic |
| 272.1353 | C10H18N5O4 | 11.0 | Metformin C6H6O4 conjugate [(α/β-ketoadipate –H2O)?]  Isomer I | 3 | Metformin | Antidiabetic |
| 272.1353 | C10H18N5O4 | 17.2 | Metformin C6H6O4 conjugate [(α/β-ketoadipate –H2O)?]  Isomer II | 3 | Metformin | Antidiabetic |
| 290.1459 | C10H20N5O5 | 17.1 | Metformin C6H8O5 conjugate [(D-glucono-δ-lactone –H2O)?] | 3 | Metformin | Antidiabetic |
| *228.1455* | C9H18N5O2 | 14.8 | Metformin C6H6O4 conjugate [(α/γ-ketovalerate –H2O)?]  Isomer I  *In network: Metformin*  *(Massbank)* | 3 | Metformin | Antidiabetic |
| 228.1455 | C9H18N5O2 | 19.5 | Metformin C6H6O4 conjugate [(α/γ-ketovalerate –H2O)?]  Isomer II | 3 | Metformin | Antidiabetic |
| 232.1343 | C8H18N5O3 | 16.6 | Metformin C4H6O3 conjugate [(small organic acid –H2O)?] | 3 | Metformin | Antidiabetic |
| *202.1299* | C7H16N5O2 | 17.2 | Metformin C3H4O2 conjugate [(lactate –H2O)?]  *In network: Metformin*  *(Massbank)* | 3 | Metformin | Antidiabetic |
| 396.1349* | ? | 15.6 | Metformin related mtb | 4 | Metformin | Antidiabetic |
| 679.2578 | C30H43N6O8S2 | 4.6 | [2M+H]^+^ of C15H22N3O4S | 3 | Sulfonylurea drug | Sulfonylurea (antidiabetic) |
| 516.1646 | C21H30N3O10S | 7.5 | Glucuronide of C15H22N3O4S | 3 | Sulfonylurea drug | Sulfonylurea (antidiabetic) |
| 604.2436* | ? | 4.8 | C15H22N3O4S related mtb | 4 | Sulfonylurea drug | Sulfonylurea (antidiabetic) |
| 338.1169 | C15H20N3O4S | 4.2 | [C15H22N3O4S –2H] | 3 | Sulfonylurea drug | Sulfonylurea (antidiabetic) |
| 324.1376 | C15H22N3O3S | 3.9 | [C15H22N3O4S –O] | 3 | Sulfonylurea drug | Sulfonylurea (antidiabetic) |
| 340.1326 | C15H22N3O4S | 4.6 | C15H22N3O4S – parent drug?  Sulfonylurea group fragments 🡪 C7H7,C7H7O2S, with ‘R’ on aromatic ring equals CH3. | 3 | Sulfonylurea drug | Sulfonylurea (antidiabetic) |
| *232.0274* | C8H10NO5S | 6.7 | Paracetamol sulfate  *In network: paracetamol*  *Massbank* | 2 | Paracetamol | Pain reliever |
| 328.1027 | C14H18NO8 | 8.7 | Paracetamol glucuronide | 2 | Paracetamol | Pain reliever |
| 313.0853 | C13H17N2O5S | 6.9 | Paracetamol N-acetylcysteine conjugate (also known as paracetamol mercapture) | 3 | Paracetamol mercapurates | Pain reliever byproduct |
| 271.0747 | C11H15N2O4S | 8.4 | Paracetamol cysteine conjugate | 3 | Paracetamol mercapurates | Pain reliever byproduct |
| 447.1068 | C17H23N2O10S | 10.1 | Paracetamol cysteine conjugate glucuronide | 3 | Paracetamol mercapurates | Pain reliever byproduct |
| 329.0802 | C13H17N2O6S | 7.4 | Paracetamol C5H9NO4S conjugate (N-carboxymethylcysteine?)  Isomer I | 3 | Paracetamol mercapurates | Pain reliever byproduct |
| 329.0802 | C13H17N2O6S | 7.9 | Paracetamol C5H9NO4S conjugate (N-carboxymethylcysteine?)  Isomer II | 3 | Paracetamol mercapurates | Pain reliever byproduct |
| 330.1118 | C13H20N3O5S | 7.1 | Paracetamol C5H10N2O3S conjugate (L-cysteinylglycine?) | 3 | Paracetamol mercapurates | Pain reliever byproduct |
| 270.0431 | C11H12NO5S | 7.0 | Paracetamol C3H4O3S conjugate (3-mercaptopuruvate?) | 3 | Paracetamol mercapurates | Pain reliever byproduct |
| 351.0315 | C11H15N2O7S2 | 9.7 | Paracetamol cysteine conjugate sulfated | 3 | Paracetamol mercapurates | Pain reliever byproduct |
| 358.1431 | C15H24N3O5S | 5.2 | Paracetamol cysteine-with-C4H9NO-adduct conjugate | 3 | Paracetamol mercapurates | Pain reliever byproduct |
| 393.0421 | C13H17N2O8S2 | 10.2 | Paracetamol N-acetylcysteine conjugate sulfated | 3 | Paracetamol mercapurates | Pain reliever byproduct |
| 459.1544 | C18H27N4O8S | 7.0 | Paracetamol N-acetylcysteine and glutamine conjugate | 3 | Paracetamol mercapurates | Pain reliever byproduct |
| 325.1910 | C20H25N2O2 | 6.9 | Quinidine | 2 (MzCloud) | Quinidine | antiarrhythmic agents (class I) |
| 327.2067 | C20H27N2O2 | 10.6 | Quinidine reduced (+2H) | 3 | Quinidine | antiarrhythmic agents (class I) |
| 341.1860 | C20H25N2O3 | 6.8 | Quinidine hydroxylated | 3 | Quinidine | antiarrhythmic agents (class I) |
| 359.1965 | C20H27N2O4 | 10.3 | Quinidine reduced (+2H) dihydroxylated | 3 | Quinidine | antiarrhythmic agents (class I) |
| 355.1652 | C20H23N2O4 | 4.8 | Quinidine oxidated (-2H) dihydroxylated  Isomer I | 3 | Quinidine | antiarrhythmic agents (class I) |
| 355.1652 | C20H23N2O4 | 6.8 | Quinidine oxidated (-2H) dihydroxylated  Isomer II | 3 | Quinidine | antiarrhythmic agents (class I) |
| 375.1914 | C20H27N2O5 | 10.3 | Quinidine reduced (+2H) trihydroxylated | 3 | Quinidine | antiarrhythmic agents (class I) |
| 345.1809 | C19H25N2O4 | 12.1 | Quinidine related mtb | 4 | Quinidine | antiarrhythmic agents (class I) |
| 357.1809 | C20H25N2O4 | 6.9 | Quinidine dihydroxylated  Isomer I | 3 | Quinidine | antiarrhythmic agents (class I) |
| 357.1809 | C20H25N2O4 | 8.0 | Quinidine dihydroxylated  Isomer II | 3 | Quinidine | antiarrhythmic agents (class I) |
| 396.0682 | C16H18N3O5S2 | 3.9 | C9H11NOS based mtb – coupled to C8H10N2 – sulphated | 4 | Unknown | Unknown |
| 492.1435 | C22H26N3O8S | 4.8 | C9H11NOS based mtb – coupled to C8H10N2 – glucuronidated | 4 | Unknown | Unknown |
| 522.1541 | C23H28N3O9S | 4.7 | C9H11NOS based mtb – coupled to C8H8N2O2 – glucuronidated – isomer I | 4 | Unknown | Unknown |
| 522.1541 | C23H28N3O9S | 4.6 | C9H11NOS based mtb – coupled to C8H8N2O2 – glucuronidated – isomer II | 4 | Unknown | Unknown |
| 506.1492 | C23H28N3O8S | 4.4 | C9H11NOS based mtb – coupled to C8H8N2O – glucuronidated | 4 | Unknown | Unknown |
| 412.0631 | C16H18N3O6S2 | 3.7 | C9H11NOS based mtb – coupled to C7H6N2O2 – sulphated | 4 | Unknown | Unknown |
| 426.0788 | C17H20N3O6S2 | 3.9 | C9H11NOS based mtb – coupled to C8H8N2O2 – sulphated | 4 | Unknown | Unknown |
| 546.1150 | C21H26N2O13S | 4.5 | C15H17N2O7S (C7H6N2O and C8H12O6S major two fragments) related mtb –  glucuronidated | 4 | Unknown | Unknown |
| 450.0397 | C15H18N2O10S2 | 3.6 | C15H17N2O7S (C7H6N2O and C8H12O6S major two fragments) related mtb –  sulphated | 4 | Unknown | Unknown |
| 370.0829 | C15H18N2O7S | 4.4 | C15H17N2O7S (C7H6N2O and C8H12O6S major two fragments) mtb | 4 | Unknown | Unknown |
| 466.0347 | C15H18N2O11S2 | 3.5 | C15H17N2O7S (C7H6N2O and C8H12O6S major two fragments) related mtb –  Sulphated and hydroxylated on C7H6N2O part | 4 | Unknown | Unknown |
| 268.xxxx* | ? | 4.3 | Shows C8H12SO6 fragment, and C7H6N2O related fragments – unclear what parent ion is. | 4 | Unknown | Unknown |

For each metabolite, the theoretical mass ([M+H ]^+^), elemental formula ([M+H ]^+^), retention time, annotation (metabolite description and further information if available), metabolomics standards initiative metabolite identification (MSI MI) level, the parent drug of the drug metabolite, and its drug class are recorded. The annotations contain references to metabolites found in literature and the MSI MI level contains information to which spectral database a spectral match was found. Masses in italic were annotated in the network, their network annotation are also given in the table.
